# Risk of fracture in adults with type 2 diabetes in Sweden: A national cohort study

**DOI:** 10.1371/journal.pmed.1004172

**Published:** 2023-01-26

**Authors:** Kristian F. Axelsson, Henrik Litsne, Konstantina Kousoula, Stefan Franzén, Björn Eliasson, Mattias Lorentzon

**Affiliations:** 1 Sahlgrenska Osteoporosis Centre, Institute of Medicine, Sahlgrenska Academy, University of Gothenburg, Gothenburg, Sweden; 2 Region Västra Götaland, Närhälsan Norrmalm Health Centre, Skövde, Sweden; 3 Region Västra Götaland, Department of Specialist Medicine, Sahlgrenska University Hospital, Gothenburg, Sweden; 4 Health Metrics Unit, Sahlgrenska Academy, University of Gothenburg, Sweden; 5 Region Västra Götaland, Center for Registries, Gothenburg, Sweden; 6 Mary McKillop Institute for Health Research, Australian Catholic University, Melbourne, Australia; 7 Region Västra Götaland, Geriatric Medicine, Sahlgrenska University Hospital, Mölndal, Sweden; Shanghai Jiao Tong University Affiliated Sixth People’s Hospital, China

## Abstract

**Background:**

Type 2 diabetes mellitus (T2DM) is considered a risk factor for fracture but the evidence regarding the impact of T2DM on fracture risk is conflicting. The objective of the study was to determine if patients with T2DM have increased fracture risk and if T2DM-related risk factors could be identified.

**Methods and findings:**

In this national cohort study in Sweden, we investigated the risk of fracture in 580,127 T2DM patients, identified through the national diabetes register including from both primary care and hospitals, and an equal number of population-based controls without diabetes matched for age, sex, and county from 2007 to 2017. The mean age at entry was 66.7 years and 43.6% were women. During a median follow-up time of 6.6 (interquartile range (IQR) 3.1 to 9.8) years, patients with T2DM had a marginally but significantly increased risk of major osteoporotic fracture (MOF) (hazard ratio (HR) 1.01 (95% confidence interval [CI] 1.00 to 1.03)) and hip fracture (HR 1.06 (95% CI 1.04 to 1.08)) compared to controls, associations that were only minimally affected (HR 1.05 (95% CI 1.03 to 1.06) and HR 1.11 (95% CI 1.09 to 1.14), respectively) by multivariable adjustment (age, sex, marital status, and an additional 20 variables related to general morbidity, cardiovascular status, risk of falls, and fracture). In a multivariable-adjusted Cox model, the proportion of the risk for all fracture outcomes (Heller’s R2) explained by T2DM was below 0.1%. Among the T2DM patients, important risk factors for fracture were a low BMI (<25 kg/m^2^), long diabetes duration (≥15 years), insulin treatment, and low physical activity. In total, 55% of the T2DM patients had none of these risk factors and a significantly lower fracture risk than their respective controls. The relatively short mean duration of T2DM and lack of bone density data, constitute limitations of the analysis.

**Conclusion:**

In this study, we observed only a marginally increased fracture risk in T2DM, a condition that explained less than 0.1% of the fracture risk. Consideration of the herein identified T2DM-related risk factors could be used to stratify T2DM patients according to fracture risk.

## Abstract 2

Kristian F. Axelsson and colleagues investigate fracture risk in patients with type 2 diabetes and seek to identify relevant risk factors.

## Author summary

**Why was this study done?:**

- Type 2 diabetes mellitus (T2DM) is considered a risk factor for fracture but the evidence regarding the impact of T2DM on fracture risk is conflicting.
- We sought to determine if patients with T2DM had increased fracture risk and if so, to identify T2DM-related risk factors.

**What did the researchers do and find?:**

- We used a national register covering nearly all adult patients with T2DM in Sweden to compare the risk of fracture between T2DM patients (*n* = 580,127) and population controls (*n* = 580,127) without T2DM.
- In general, the risk of fracture was only marginally increased (by 1% for major osteoporotic fracture (MOF) and by 6% for hip fracture) for patients with T2DM compared to controls, but for patients with presence of risk factors, such as low BMI, long T2DM duration, insulin treatment, and/or low physical activity, the risk increase was more substantial (20% or higher) and potentially clinically relevant.

**What do these findings mean?:**

- Our findings suggest that T2DM per se should not be considered an important risk factor for fracture.
- Special consideration of the T2DM-related risk factors can be used to identify T2DM patients with a clinically relevant increased fracture risk.
- Further studies are needed to develop fracture risk calculators for different settings and populations for T2DM patients specifically.

## Introduction

It has been estimated that 151 million people have type 2 diabetes mellitus (T2DM) globally and that the prevalence is expected to increase to 324 million by 2030 [1]. T2DM may result in many deleterious complications, of which the most severe include cardiovascular and renal [2]. Peripheral neuropathy, visual impairment, foot ulcers, orthostatic hypotension, and impaired physical function contribute to the increased risk of falls in T2DM [3–6].

A meta-analysis of cohort and case-control studies, including over 17.5 million participants, from 2020 found that the risk of hip fracture was increased by 1.13 times for men and 1.34 times for women in patients with T2DM, while the risk for nonvertebral fracture was increased by 19% in combined dataset of men and women [7]. Longer diabetes duration and insulin use were associated with a higher risk. A recent, large Swedish cohort study found that T2DM patients without medication or using oral antidiabetic treatment had neutral or reduced risk, respectively, and only those with insulin treatment had an increased fracture risk [3]. Thus, the risk increase seen in T2DM patients seems to be dependent on T2DM-related risk factors and not universal for the patient population.

The risk of hip fracture increases with declining bone mineral density (BMD) [8]. It is the most severe osteoporotic fracture and frequently results in functional decline, increased risk of additional fractures, as well as increased morbidity and mortality [9,10]. Patients with T2DM have higher than normal or normal BMD, possibly due to hyperinsulinemia, but seem to fracture at higher BMD than those without diabetes, indicating other mechanisms of bone fragility in T2DM [11,12]. Based on these data, it has been argued that other risk factors than those included in the globally most widely used fracture prediction assessment tool FRAX, contribute to fracture risk in T2DM [4,13]. Affected bone material properties, due to increased glycation of bone matrix proteins, has been proposed as a potential contributor to the increased fracture risk [4,14].

As a probable result of improved risk factor control, including blood glucose lowering therapies, use of statins, treatment of hypertension, and lifestyle interventions, such as smoking cessation and physical activity, the management of T2DM has improved tremendously in Europe and North America in the past few decades, leading to reductions in diabetes-related complications and mortality [15–18]. We therefore hypothesized that the generally accepted notion of T2DM per se, being a clinically relevant risk factor for fracture may not be valid in modern management of the disease.

The primary objective of this study was to determine if patients with T2DM have increased fracture risk compared to matched controls, using a nationwide Swedish cohort. In ancillary analyses, we sought to identify independent diabetes-related risk factors for fracture using machine learning, to distinguish a possible subgroup of T2DM patients with a clinically relevant risk increase.

## Methods

### Study design and data sources

To compare the risk of fractures between persons with T2DM and matched controls, this retrospective cohort study used national medical registers in Sweden. The study was funded by the Swedish Research Council and the Sahlgrenska University Hospital without industry support and approved by the Swedish Ethical Review Authority (2022-00796-01), which allowed the presented research without the need for informed consent, due to the use of anonymized study participant information. This study is reported as per the Strengthening the Reporting of Observational Studies in Epidemiology (STROBE) guideline (STROBE checklist in S1 Appendix).

The Swedish National Diabetes Register (NDR) started in 1996, includes information on risk factors and complications of diabetes and covers 88% of all patients with diabetes in Sweden [19,20]. T2DM was defined according to epidemiologic criteria, i.e., treatment with diet, oral antihyperglycemic agents, insulin, or a combination thereof. If treated with insulin, only patients 40 years of age or older at the time of diabetes diagnosis were included. Hospital-based diagnoses (from both inpatient and outpatient visits) were retrieved from the National Patient Register and data on socioeconomics and death from Statistics Sweden. The Swedish Prescribed Drug Register, starting July 1, 2005, was used to collect data on medications. Linkage between the registers was enabled using the personal identification number, assigned to all inhabitants in Sweden at birth or at the time of immigration (S2 Appendix).

Baseline was defined as the first date of registration in NDR, but not earlier than January 1, 2007, to guarantee a minimum of 1 year medication history for all patients. Each case, patient was assigned 1 control from the general population matched on birth year, sex, and county using replacement to avoid bias [21]. The population controls were assigned the same baseline date as their corresponding case and only controls without diabetes at baseline were selected.

### Outcomes

Fracture outcomes, injurious falls, and deaths were assessed. Any fracture included all non-pathological fracture diagnoses regardless of type of trauma (head and phalangeal fractures excluded). Major osteoporotic fractures (MOFs) included fractured hip, vertebrae, proximal humerus, wrist, and pelvis. Hip fracture included fractures of the femoral head, neck, trochanter or subtrochanteric part of the femur accompanied with a code for surgical procedure. Injurious falls were defined as any hospital event with a code for injury and fall, but without a fracture code. The specific codes are presented in S1 Table.

### Baseline data

A large number of covariates representing prevalent illnesses and prescribed medications with potential impact on an included individual’s comorbidity and risk of fracture were selected (S2 Table). The Charlson comorbidity index was calculated to summarize and quantify comorbidity [22]. Prevalent medication variables included the last 12 month’s prescriptions from both hospitals and primary care. Descriptive data with diabetes parameters for the T2DM patients was collected from NDR at baseline or up to 1 year prior to the inclusion.

### Statistical analyses

Descriptive baseline statistics are presented in terms of counts with percentage for categorical variables, averages with standard deviations (SDs) for normally distributed continuous variables and medians with interquartile range (IQR) for other continuous variables. Standardized mean differences (SMDs) were calculated to present differences in baseline characteristics between the T2DM patients and the controls (S2 Appendix). Event rates were calculated as the number of events per 1,000 person-years and are presented with exact Poisson 95% confidence intervals (CIs). The cumulative incidence of events was estimated using 1 minus the Kaplan–Meier estimate of the corresponding survival function and presented with 95% CIs. Yearly incident rates were estimated as the number of events occurring during each year divided by the number of person-years accumulated during each year, standardized to the age and sex distribution in the entire cohort and presented as event rates per 1,000 person years with 95% CIs based on a normal approximation accounting for the weights.

Cox regression models were used to calculate hazard ratios (HRs), both unadjusted with T2DM or control as the only independent variable as well as with extensive multivariable adjustment (not including diabetes treatment). The follow-up time was censored for end of study (December 31, 2017), emigration, and death. Furthermore, the controls were also censored for diabetes, i.e., NDR registration, diagnosis, or prescription. The Cox assumption of proportional hazards was tested using graphical methods. The standard errors in the Cox regression were estimated using a robust sandwich estimator accounting for the selection of controls with replacement. To estimate the importance of each variable included in the multivariable-adjusted Cox model, Heller’s R2 was used and R2 values are presented graphically for fracture outcomes [23]. Interactions were tested using fully adjusted Cox models, with interaction terms for the group variable (T2DM) and sex, age, Charlson comorbidity index and previous fracture, respectively. For analysis of interaction, *p*-values less than 0.10 were considered significant.

To assess the potential impact of death as a competing risk, the cumulative incidence function, or subdistribution function, of fractures with death as competing risk was estimated using the Aalen–Johansen estimator [24]. Also, for a subset of 50.000 randomly selected persons, the subdistribution hazard for fracture was compared between persons with T2DM and controls using a Fine and Grey model with death as the competing risk [25].

In order to identify diabetes-related factors independently associated with fracture risk, the variables from the Swedish National Diabetes Register were first imputed using MICE (S1a–S1i Fig). Machine learning using Gradient Boosting Machines with interaction depth of 2, thus including all pairwise interactions, was then applied to a model including all variables in Table 1, both general comorbidity and fracture risk factors as well as the diabetes-related variables (imputed) [26].

**Table 1:** Baseline characteristics of patients with type 2 diabetes and controls.

	**Controls**	**T2DM**	
**A. Information from National Registers**	***N* = 580,127**	***N* = 580,127**	**SMD** *
Age, years	66.70±11.92	66.70 ± 11.92	<0.001
Female sex, *n* (%)	253,026 (43.6)	253,026 (43.6)	0
Sickness benefits, *n* (%)	30,393 (5.2)	46,573 (8.0)	0.112
Marital status			0.065
Married, *n* (%)	319,840 (55.1)	301,037 (51.9)	
Unmarried, *n* (%)	86,858 (15.0)	92,647 (16.0)	
Divorced, *n* (%)	94,934 (16.4)	101,823 (17.6)	
Widow(er), *n* (%)	78,495 (13.5)	84,620 (14.6)	
Urban residency, (>200 per km^2^), *n* (%)	148,487 (25.6)	143,559 (24.7)	0.020
Non-Nordic citizenship at birth, *n* (%)	41,591 (7.2)	69,396 (12.0)	0.164
Charlson comorbidity index			0.243
0, n (%)	445,415 (76.8)	386,608 (66.6)	
1–2, n (%)	108,738 (18.7)	151,920 (26.2)	
≥3, n (%)	25,974 (4.5)	41,599 (7.2)	
Osteoporosis diagnosis, *n* (%)	7,502 (1.3)	5,447 (0.9)	0.034
Conditions associated with osteoporosis, *n* (%)^†^	4,768 (0.8)	6,954 (1.2)	0.038
Alcohol related disease, *n* (%)	8,266 (1.4)	11,813 (2.0)	0.047
Rheumatoid arthritis, *n* (%)	6,992 (1.2)	7,484 (1.3)	0.008
Osteoporosis medication, *n* (%)	19,591 (3.4)	16,290 (2.8)	0.033
Calcium + Vitamin D, *n* (%)	22,547 (3.9)	21,343 (3.7)	0.011
Oral prednisolone, *n* (%)	28,826 (5.0)	36,795 (6.3)	0.059
Prevalent fracture, *n* (%)	79,312 (13.7)	77,173 (13.3)	0.011
Prevalent fall injury, *n* (%)	50,390 (8.7)	55,282 (9.5)	0.029
Nitrates, *n* (%)	17,717 (3.1)	37,449 (6.5)	0.160
Diuretics, *n* (%)	51,573 (8.9)	109,130 (18.8)	0.290
Thiazides, *n* (%)	22,674 (3.9)	36,914 (6.4)	0.111
Beta blockers, *n* (%)	111,118 (19.2)	208,867 (36.0)	0.384
Calcium channel blockers, *n* (%)	61,147 (10.5)	125,180 (21.6)	0.304
RAS inhibitors, *n* (%)	107,405 (18.5)	259,408 (44.7)	0.587
Statins, *n* (%)	78,835 (13.6)	208,273 (35.9)	0.535
T2DM medications any, *n* (%)	0 (0.0)	360,118 (62.1)	1.809
Insulin, *n* (%)	0 (0.0)	108,483 (18.7)	0.678
Metformin, *n* (%)	0 (0.0)	270,639 (46.7)	1.322
Sulfonylureas, *n* (%)	0 (0.0)	77,611 (13.4)	0.556
DPP-4 inhibitors, *n* (%)	0 (0.0)	7,222 (1.2)	0.159
GLP-1 analogues, *n* (%)	0 (0.0)	1,440 (0.2)	0.071
SGLT2 inhibitors, *n* (%)	0 (0.0)	590 (0.1)	0.045
Glitazones, *n* (%)	0 (0.0)	9,647 (1.7)	0.184

**B. Information from Swedish National Diabetes Register**	**Controls**	**T2DM**	**N (%)** ^**‡**^
BMI, kg/m^2^	–	30.1 ± 5.5	415,983 (72)
Normal/underweight (<25), *n* (%)	–	66,425 (16.0)	
Overweight (25–29.9), *n* (%)	–	161,328 (38.8)	
Obesity class I (30–34.9), *n* (%)	–	118,394 (28.5)	
Obesity class II (≥35), *n* (%)	–	69,836 (16.8)	
Systolic blood pressure, mmHg	–	137.8±17.1	454,810 (78)
Diastolic blood pressure, mmHg	–	78.5±10.1	454,252 (78)
Glycated hemoglobin (HbA1c)			474,337 (82)
mmole/mole	–	54.3 ± 16.0	
%	–	7.1 ± 1.5	
Cholesterol, total, mmole/liter	–	5.0 ± 1.1	364,565 (63)
Age at diagnosis of diabetes, years	–	61.4 ± 11.9	537,739 (93)
Median duration of diabetes at baseline (IQR), years	–	2 (0–8.6)	537,739 (93)
Current smoking–no (%)	–	62,035 (15.4)	403,088 (69)
Physical activity–no (%)^**§**^			345,218 (60)
Never	–	53,782 (15.6)	
<1 per week	–	46,260 (13.4)	
1–2 per week	–	69,388 (20.1)	
3–5 per week	–	74,608 (21.6)	
Daily	–	101,180 (29.3)	
Chronic kidney disease (renal failure)–no (%)			423,611 (73)
No (GFR ≥60)		351,508 (83.0)	
Moderate (GFR 30–59.9)		67,710 (16.0)	
Severe (GFR 15–29.9)		3,811 (0.9)	
Terminal (GFR <15)		582 (0.1)	

Baseline characteristics of patients with type 2 diabetes (T2DM) and population controls without diabetes, matched according to birth year, sex, and county. Part A of the table uses information from national registers covering all swedes, thus the information is available for both T2DM patients and controls. Part B of the table includes information from the Swedish National Diabetes Register and is only available for the T2DM case patients. Values are presented as mean ± SD, if not otherwise indicated. The historic window was since 1998 for fracture and fall, 5 years for other diagnoses and 1 year for medications. For detailed definitions of covariables, see S2 Table.

* SMD, see S1 Appendix for formulas. All *p*-values (except for age and sex) were <0.001.

† Conditions associated with osteoporosis = hyperthyroidism, hypogonadism, malnutrition, osteogenesis imperfecta, chronic liver disease, hyperparathyroidism.

‡ *N* (%) = number of T2DM patients with available values and percentage of all T2DM patients (580,127).

§ Physical activity = 30 minutes’ walk or equivalent.

IQR, interquartile range; SMD, standardized mean difference; T2DM, type 2 diabetes mellitus.

As a result of the observed weak association between T2DM and fracture risk as well as the relatively weak associations with the top T2DM related variables, stratification of the heterogenous T2DM patients was performed using the diabetes-related variables that had a relative importance of at least 1% for any fracture. Thresholds for each respective risk variable were deemed clinically relevant and selected based on an observed increased fracture risk of at least 20%, compared to age, sex, and county-matched controls. Also, in response to reviewer feedback, analyses results regarding the association between T2DM and incident injurious falls were added. Statistical analyses were performed using SPSS 28.0.1.0, R 4.02, and R-Studio version 1.4.1106.

## Results

### Study population

A total of 580,127 T2DM patients and an equal number of matched controls were included. Baseline characteristics are presented in Table 1. The mean age at entry was 66.7 years and 43% were women. The mean glycated hemoglobin level was 7.1% (54.3 millimole per mole). At baseline (first register entry), the median time since diagnosis of diabetes was 2.0 (IQR 0 to 8.6) years. The prevalence of hypertensive medications and statins was more common among the T2DM patients than among the controls, whereas the traditional risk factors for fracture were similar in prevalence between the 2 groups. The patients were followed for a median time of 6.6 (IQR 3.1 to 9.8) years.

### Risk of fractures

**Fig 1:**
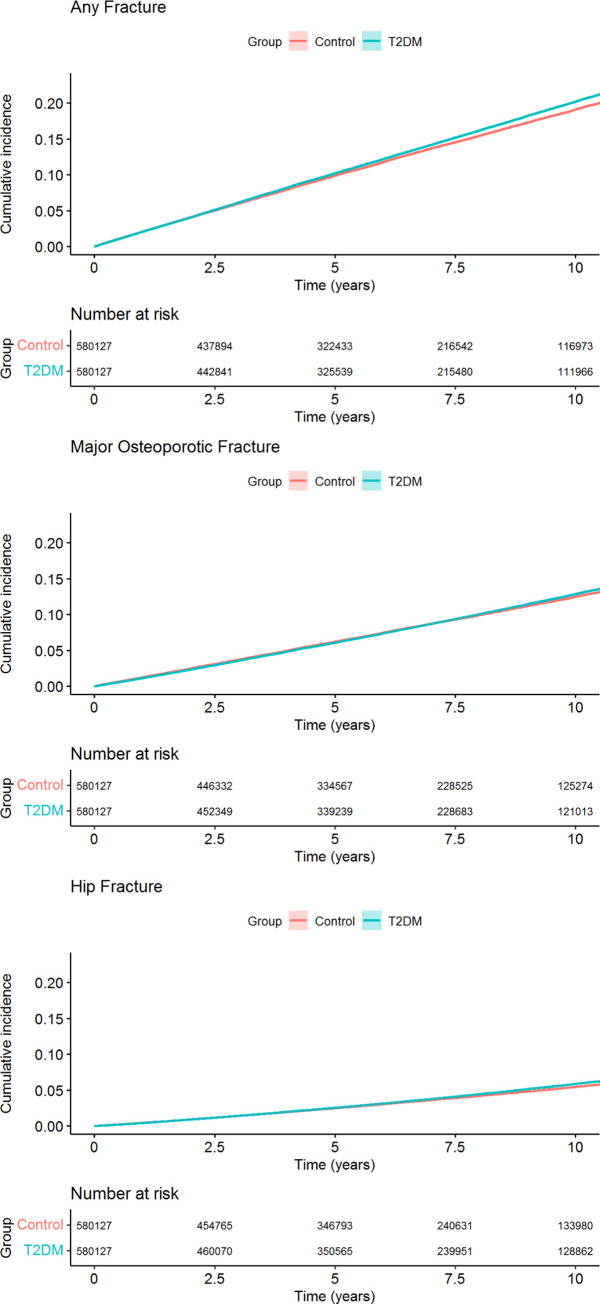
Cumulative incidence of fractures for T2DM patients and controls. The cumulative incidence of events was estimated using 1 minus the Kaplan–Meier estimate of the corresponding survival function and presented with 95% CIs. In the above graphs, both the estimates and the 95% CIs are plotted. CI, confidence interval; T2DM, type 2 diabetes mellitus.

There were 75,502 (13.0%) T2DM patients with fractures during follow-up compared to 71,546 (12.3%) among the controls translating to incidence rates of 22.2 (22.0 to 22.3) and 21.1 (20.9 to 21.2) per 1,000 person-years, respectively. Patients with T2DM had a small but statistically significantly increased risk of any fracture (HR 1.05 (95% CI) 1.04 to 1.06), MOF (HR 1.01 (1.00 to 1.03)), and hip fracture (HR 1.06 (1.04 to 1.08)), compared to controls (Fig 1), associations that were only marginally affected by multivariable adjustment (Table 2). The yearly incidence rates from 2007 to 2017, standardized for age and sex, were similar in T2DM patients and controls, respectively (S2 Fig). There were significant interactions between the group variable and sex, age, and Charlson comorbidity index, respectively, but not for previous fracture, although the differences in associations were clinically modest (S3 Fig). The risk of fracture compared to controls was a little higher in men than women, also after adjustment (S3 Table). While there were slightly more male smokers among the T2DM patients, the men were also less underweight, more physically active, and had better kidney function (S4 Table). Differences in fracture risk between T2DM patients and controls were only seen in patients with a Charlson comorbidity index of 2 or lower and in those younger than 80 years (S3 Fig). Compared to matched controls, men with T2DM had a higher fracture risk than women (S3 Fig and S3 Table). Furthermore, we observed an increased risk of lower leg fractures and proximal humerus fracture, while the risk of wrist fracture was lower in T2DM patients compared to controls (S5 Table).

**Table 2:** Fracture outcomes in patients with type 2 diabetes and controls.

	Controls	T2DM
*N* =	580,127	580,127
Time at risk, years median (IQR)	6.6 (3.0–9.8)	6.6 (3.1–9.7)
**Any fracture**		
*n* (%)	71,546 (12.3%)	75,502 (13.0%)
Rate, per 1,000 person-years	21.1 (20.9–21.2)	22.2 (22.0–22.3)
Cox, unadjusted, HR (95% CI)	Ref. [1]	1.05 (1.04–1.06)
Cox, adjusted, HR (95% CI)	Ref. [1]	1.07 (1.05–1.08)
**Major osteoporotic fracture**		
*n* (%)	46,027 (7.9%)	46,895 (8.1%)
Rate, per 1,000 person-years	13.2 (13.0–13.3)	13.3 (13.2–13.4)
Cox, unadjusted, HR (95% CI)	Ref. [1]	1.01 (1.00–1.03)
Cox, adjusted, HR (95% CI)	Ref. [1]	1.05 (1.03–1.06)
**Hip fracture**		
*n* (%)	19,497 (3.4%)	20,705 (3.6%)
Rate, per 1,000 person-years	5.4 (5.3–5.5)	5.7 (5.7–5.8)
Cox, unadjusted, HR (95% CI)	Ref. [1]	1.06 (1.04–1.08)
Cox, adjusted, HR (95% CI)	Ref. [1]	1.11 (1.09–1.14)
**Death**		
*n* (%)	104,145 (17.9%)	145,228 (25.0%)
Rate, per 1,000 person-years	28.5 (28.3–28.7)	39.6 (39.4–39.8)
Cox, unadjusted, HR (95% CI)	Ref. [1]	1.39 (1.38–1.40)
Cox, adjusted, HR (95% CI)	Ref. [1]	1.31 (1.30–1.32)

Outcomes for patients with type 2 diabetes and population controls without diabetes, matched according to birth year, sex, and county. Event rates were calculated as the number of persons with respective outcome per 1,000 person-years and are presented with exact Poisson 95% CIs. The adjusted Cox model is adjusted for age, sex, sickness benefits, marital status, urban residency, non-Nordic citizenship at birth, Charlson comorbidity index, osteoporosis diagnosis, conditions associated with osteoporosis, alcohol-related disease, rheumatoid arthritis, osteoporosis medication, calcium + vitamin D use, oral prednisolone medication use, prevalent fracture, prevalent fall injury, nitrates, diuretics, thiazides, beta blockers, calcium channel blockers, RAS inhibitors, and statins.

CI, confidence interval; HR, hazard ratio; IQR, interquartile range; RAS, renin–angiotensin system; T2DM, type 2 diabetes mellitus.

### Analysis of variable importance of risk factors

**Fig 2:**
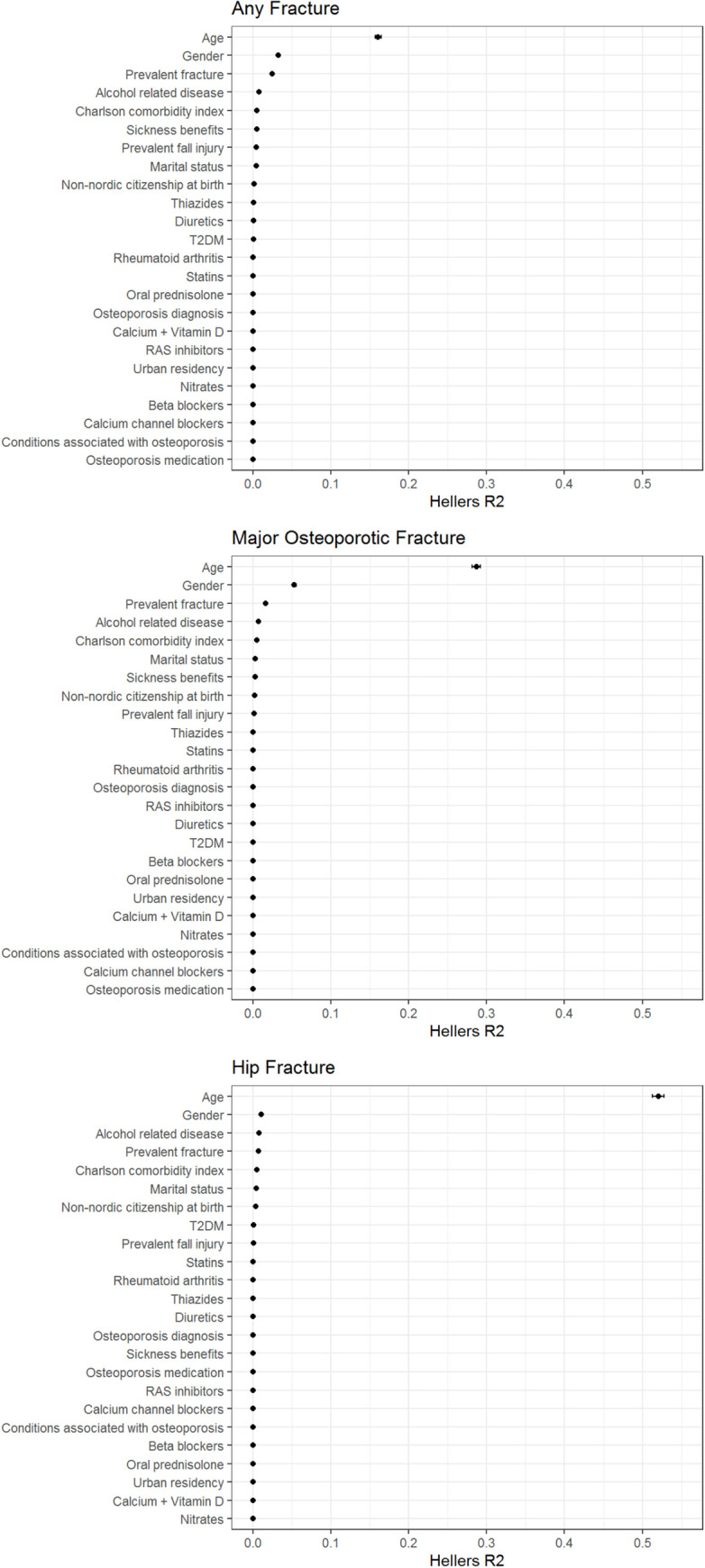
Variable importance of risk factors predicting fractures. To estimate the importance of each variable included in the multivariable-adjusted Cox model, Heller’s R2 was used. R2 values for all covariates in the model are presented graphically. The sum of all the attributable factors was 0.26, 0.38, 0.56 for any, major osteoporotic and hip fracture, respectively.

In a multivariable-adjusted Cox model, using Heller’s R2, the variable importance (R2) of T2DM was lower than 0.1% for all fracture outcomes and more than half of the other risk factors were more important, a finding which was highly consistent for all fracture outcomes (Fig 2).

### Risk of injurious falls

There were 69,089 (11.9%) patients with an injurious fall during follow-up compared to 57,952 (10.0%) among the controls translating to incidence rates of 20.1 (20.0 to 20.3) and 16.8 (16.6 to 16.9) per 1,000 person-years, respectively. Patients with T2DM had a significantly increased risk of injurious fall (HR 1.20 (1.19 to 1.22) compared to controls, an association that was slightly attenuated by multivariable adjustment (S5 Table).

### Mortality and competing risk

There were 145,228 (25.0%) deaths among the T2DM patients during follow-up compared to 104,145 (17.9%) among the controls translating to incidence rates of 39.6 (39.4 to 39.79) and 28.5 (28.3 to 28.7) per 1,000 person-years, respectively. Patients with T2DM had a significantly increased risk of death (HR 1.39 (1.38 to 1.40)) compared to controls, an association slightly attenuated by multivariable adjustment (Table 2). Visualization of the cumulative incidence functions of each outcome with death as a competing risk revealed a minimal impact on the studied associations (S4 Fig). Fine and Grey analyses with death as a competing risk demonstrated that T2DM was not associated with an increased risk of any fracture, MOF, or hip fracture (S6 Table).

### Risk stratification per number of diabetes-related risk factors

**Fig 3:**
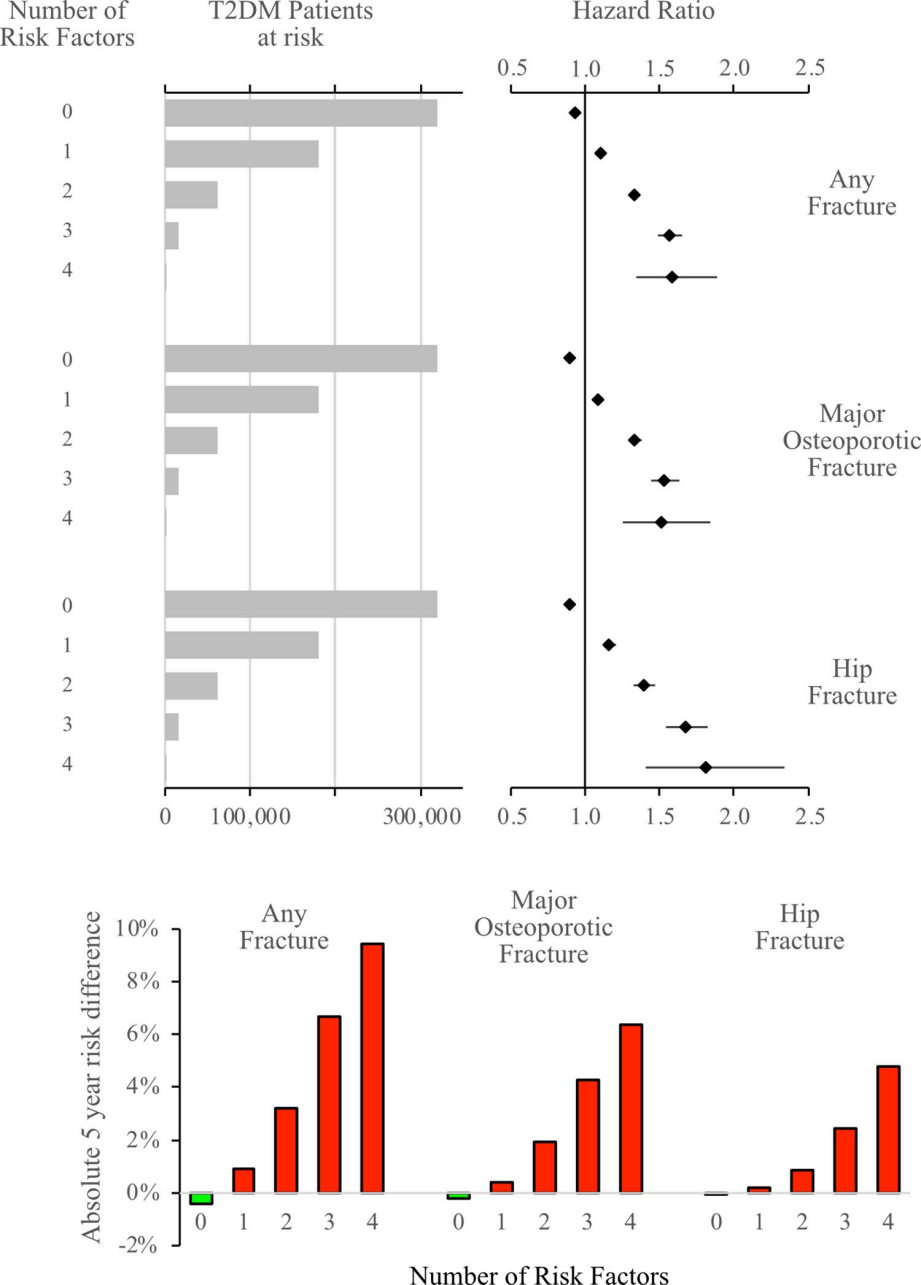
Relative and absolute risk of fracture in T2DM patients—per number of risk factors compared to matched controls. Relative and absolute risk difference in T2DM patients per number of risk factors compared to matched controls using a multivariable-adjusted Cox regression model. The 4 diabetes-related risk factors were: (1) BMI <25; (2) time since diabetes diagnosis >15 years; (3) insulin treatment; and (4) absence of physical activity. T2DM, type 2 diabetes mellitus.

To allow pairwise interaction, we used Gradient Boosting Machines, to identify the 4 most important independent diabetes-related risk factors for fracture (S5–S7 Figs). All T2DM patients were then compared to their respective controls, in a multivariable-adjusted Cox model stratified for each risk factor. By using a significant risk increase of 20% (HR of at least 1.2) as a threshold, the 4 following risk factors were defined: BMI <25 kg/m^2^, diabetes duration ≥15 years, insulin treatment the past year, and low level of physical activity (S8 Fig). For each additional present T2DM risk factor, the risk of fracture increased, and the number of patients categorized decreased (Fig 3). Altogether, only 14% of the T2DM patients had 2 or more risk factors, which corresponded to an increased fracture risk of 20% or more compared to their respective controls. The majority (55%) of the T2DM patients had none of these risk factors, and therefore a small but significantly lower fracture risk than their respective controls. The risk gradient, by number of risk factors, was more pronounced among the relatively younger T2DM patients (S9 Fig). Among the T2DM patients younger than 64 years, having 3 to 4 risk factors, the risk of hip fracture and MOF was over 6 and 2 times higher, respectively, than in the controls. Conversely, having 3 to 4 risk factors was only associated with marginally increased fracture risk in those 80 years or older (S9 Fig).

## Discussion

In this nationwide cohort study of patients with T2DM and controls, presence of T2DM was not a clinically relevant risk factor for any fracture, MOF, or hip fracture, regardless of sex or adjustment for confounders. Although the risk of these fracture outcomes was increased by 5% to 6%, the proportion of the variation in fracture risk (for all fracture outcomes) explained by T2DM was negligible, less than 0.1%, with many other risk factors, such as age, sex, and prevalent fracture, contributing substantially more. In analyses considering the competing risk of death, which was higher in T2DM, the association between T2DM and fracture risk was completely absent. Using machine learning, we identified 4 risk factors for fracture, which were used to stratify T2DM patients according to risk. Altogether, only 14% of the T2DM patients had 2 or more risk factors, which corresponded to an increased fracture risk of at least 20%, compared to controls. Although the proportion identified having an increased risk was small, the estimated number of T2DM patients with increased fracture risk globally would exceed 21 million, considering that 151 million people suffer from T2DM [1]. The majority (55%) of the T2DM patients had a small but significantly lower fracture risk than their respective controls. These findings challenge the widely accepted assumption that fracture risk is elevated in T2DM per se. As demonstrated by this analysis, evaluation of T2DM-related risk factors is needed to interrogate the heterogeneity in fracture risk seen in T2DM.

In the present analyses, the risk of injurious falls without fracture was higher among patients with type 2 diabetes. These results suggest that T2DM leads to more frequent falls, possibly due to impaired physical function, which is in agreement with previous findings [5], and may suggest that an increased risk of falls, rather than bone fragility [14], may be the most important mechanism for the previously observed increased fracture risk in T2DM [7,27,28].

In a recent meta-analysis including 10 cohort studies of T2DM patients and controls, the risk was increased by 33% and 19% for hip fracture and nonvertebral fracture, respectively [7]. Only 3 of these cohorts were of considerable size and none were nationwide. First, Rathman and colleagues investigated the risk of hip fracture and other fractures in 297,104 patients with T2DM from primary care centers in Germany and matched controls. The risk of hip fracture and any fracture was increased by 56% and 36%, respectively, but patients with previous fracture, and with diseases related to bone fragility, as well as several other comorbidities were excluded, preventing unbiased conclusions regarding fracture risk in an unselected T2DM population [29]. Second, Martinez-Laguna and colleagues studied hip fracture risk in 58,483 newly diagnosed T2DM patients and matched controls in Spain. In an unadjusted analysis, the risk of hip fracture was not significantly increased in T2DM patients (HR 1.11, 95% CI (0.99 to 1.24)) [30]. Third, in 79,159 older patients in Sweden (81 years on average) with T2DM, Wallander and colleagues concluded that the risk of hip fracture was reduced in those without diabetes medication, not significantly increased in those with oral diabetes medication, and only slightly increased in those with insulin treatment [3]. In agreement with a previously reported lower risk of wrist fracture found in T2DM patients in another Swedish cohort [3], the present analysis demonstrated a reduced risk of wrist fractures by over 20%, depending on adjustment. This finding is puzzling but can be explained by a known lower risk for wrist fracture observed in obesity, a condition more common in the T2DM population [31]. Thus, the largest cohort studies investigating fracture risk in T2DM do not consistently support that T2DM is an important risk factor for fracture.

The present study is the by far largest performed, based on an overwhelming majority of T2DM patients in Sweden, with 88% register coverage [20], providing a very robust evaluation of fracture risk in the diabetes type 2 population and in matched population controls. The risk of any fracture, MOF, and hip fracture was only marginally increased in T2DM compared to controls, with highly similar results from men and women analyzed separately. Thus, these results show that stratification of the T2DM population is necessary, and further imply that taking the herein identified risk factors (duration of T2DM, low physical activity, BMI, and insulin treatment) into account, can be used to identify T2DM patients with at least a 20% higher risk of fracture. Previous smaller studies in various countries support the heterogeneity in fracture risk observed in T2DM patients in this study. For example, in a study of 82,094 Canadian patients with diabetes (T2DM and type 1 diabetes mellitus combined), those newly diagnosed had lower risk and those with long disease duration had higher risk for osteoporotic fracture than non-diabetic controls [32]. Additionally, results from a nationwide study of diabetes patients and controls from Scotland demonstrated that the risk of hip fracture was only slightly increased in T2DM patients, and that the risk was dependent on BMI, in that T2DM patients with low BMI had increased risk and those with higher BMI had lower risk, supporting the role of BMI for fracture risk observed in the present study [33].

The present analysis has limitations. Although extensive information regarding comorbidity and medications was available on all study subjects, and the T2DM population was very well characterized with a multitude of data on clinical risk factors, levels of glycated hemoglobin, lifestyle factors, and diabetes complications, the corresponding data was not available for the controls. Data from a Canadian study of eight 676 T2DM patients (90% female) showed that T2DM was associated with higher BMI and increased risk of hip fracture and that the latter association was independent of BMD [34]. A recent population-based study of older women reported higher BMD in participants who had T2DM than in those without [14]. Thus, the protective effect of higher BMD and higher BMI [35,36], which is not adjusted for in this study, could explain the only marginal increase in T2DM patients, compared to the controls. Since no data on BMD were available in the present study, in either patients or controls, and data on BMI were only available in T2DM patients, we were unable to investigate if BMD and BMI affected the herein-studied associations. Furthermore, it should be acknowledged that as we and others have observed an increasing fracture risk with increasing T2DM duration, the relatively short mean duration of T2DM of the included patients likely weakened the association between T2DM and fracture risk in the whole cohort. Although this study is the largest yet, it investigates fracture risk in Swedish patients with T2DM only. Thus, the results may not be transferrable to other populations and countries.

The present analysis also has strengths. This is to our knowledge the by far largest cohort of well-characterized patients with T2DM ever investigated in relation to fracture risk. As a direct result of the high uptake and registration rate of 88% in the Swedish National Diabetes Register, the used nationwide cohort is highly representative of the population with T2DM in Sweden [20]. Extensive data regarding comorbidity and medication were used in multivariable adjustments allowing control of a large number of potential confounders. To avoid bias, all persons were considered and allowed as controls, until becoming diagnosed with T2DM, and controls were sampled with replacement. Extensive analyses to avoid confounding by competing risk of death were undertaken and did not change our main findings. The novel machine learning methodology applied, allowed selection of the T2DM-related risk factors of importance for fracture risk and enabled identification of a small proportion of patients with a clinically relevant increased fracture risk.

In conclusion, we observed only a marginally increased fracture risk in T2DM, a condition which explained less than 0.1% of the fracture risk. Consideration of the herein identified T2DM-related risk factors could be used to stratify T2DM patients according to fracture risk.

## Supporting information

S1 Appendix Strobe statement. (DOCX)

S2 Appendix Extended methods. Additional information on registers used. Standardized differences.
(DOCX)

S1 Fig Histograms without and with imputation. Variables from the Diabetes Register with 60% registration rate or higher were included and imputed using the MICE-package in R-Studio (Multivariate Imputation by Chained Equations), using 20 iterations with Nelson–Aalen estimates for all the outcomes. In addition to the outcomes, all the variables included in Table 1 were included in the imputation. Histograms without and with imputation are presented below for the following variables: (a) BMI (kg/m^2^). (b) Systolic blood pressure (mmHg). (c) Diastolic blood pressure (mmHg). (d) HbA1c (mmole/mole). (e) Cholesterol (mmole/liter). (f) Duration of diabetes (years). (g) Smoker (yes/no) Smoker = 1 cigarette per day or more or pipe smoker. Includes those who stopped smoking less than 3 months earlier. (h) Physical activity (30 min walk or equivalent, times per week). Groups of physical activity (30 minutes’ walk or equivalent) 1: Never, 2: <1 per week, 3: 1–2 per week, 4: 3–5 per week, 5: Daily. (i) Chronic kidney disease (renal failure), groups: No (GFR ≥60), 1: Moderate (GFR 30–59.9), 2: Severe (GFR 15–29.9), 3: Terminal (GFR <15)).
(DOCX)

S2 Fig Yearly incidence rates in T2DM patients vs. population controls. Yearly event rates were estimated as the number of events occurring during each year divided by the number of person-years accumulated during each year, standardized to the age and sex distribution in the entire cohort and presented as event rates per 1,000 person years with 95% CIs based on a normal approximation accounting for the weights.
(DOCX)

S3 Fig Multivariable-adjusted risk of any fracture in T2DM patients vs. controls–subgroup analyses per age, sex, previous fracture, and Charlson comorbidity index. *P*-values for the interaction term are stated per group. HRs for any fracture were calculated in Cox models adjusted for age, sex, sickness benefits, marital status, urban residency, non-Nordic citizenship at birth, Charlson comorbidity index, osteoporosis diagnosis, conditions associated with osteoporosis, alcohol-related disease, rheumatoid arthritis, osteoporosis medication, calcium + vitamin D use, oral prednisolone medication use, prevalent fracture, prevalent fall injury, nitrates, diuretics, thiazides, beta blockers, calcium channel blockers, renin-angiotensin system inhibitors, and statins.
(DOCX)

S4 Fig Cumulative incidence function in T2DM patients vs. population controls. The cumulative incidence function, or subdistribution function, of fracture/injurious fall with death as competing risk was estimated using the Aalen–Johansen estimator. All patients included.
(DOCX)

S5 Fig Identification of diabetes-related risk factors using Gradient Boosting Machines—all cases including imputed values. Machine learning using Gradient Boosting Machines was applied to all T2DM cases (*N* = 580,127, no controls). The settings allowed interaction depth 2, i.e., all pairwise interactions were enabled. All variables in Table 1, both general comorbidity and fracture risk factors as well as specific diabetes-related variables (imputed) were included. For any fracture, the top 4 diabetes-related variables are marked with an asterisk and percentages for relative importance included in the pie chart. The color labels are the same for all 3 outcomes.
(DOCX)

S6 Fig Identification of diabetes-related risk factors using Gradient Boosting Machines—only complete cases, no imputed values. Machine learning using Gradient Boosting Machines was applied to all T2DM cases with complete values (*N* = 209,802, no controls). The settings allowed interaction depth 2, i.e., all pairwise interactions were enabled. All variables in Table 1, both general comorbidity and fracture risk factors as well as specific diabetes-related variables were included. For any fracture, the top 4 diabetes-related variables are marked with an asterisk and percentages for relative importance included in the pie chart. The color labels are the same for all 3 outcomes.
(DOCX)

S7 Fig Analysis of the top 4 variables independent association to any fracture. All the T2DM cases (without controls) were included in a Cox regression model, fully adjusted and also including the variables from the Diabetes Register, to investigate the risk of any fracture. Imputed values from the Diabetes Register were included; the continuous variables were splined with 5 degrees of freedom. The figures illustrate the association between the top 4 covariates and any fracture with their respective histograms included. All values are from the same regression model. Numbers of T2DM patients are indicated on the right y-axis and the HRs on the left y-axis.
(DOCX)

S8 Fig Comparison of T2DM patients to population controls—per risk factor. (a) Risk of any fracture among T2DM patients compared to matched population controls. The analysis was stratified for BMI. Values of BMI were rounded off to integers. The risk of any fracture was analyzed using multivariable-adjusted Cox models. A 20% risk increase (HR 1.20) was used as a clinically relevant threshold. Number of cases/controls in each group is indicated on the left y-axis and the HRs on the right y-axis. (b) Risk of any fracture among T2DM patients compared to matched population controls. The analysis was stratified for duration of diabetes. Values of duration were rounded off to integers. The risk of any fracture was analyzed using multivariable-adjusted Cox models. A 20% risk increase (HR 1.20) was used as a clinically relevant threshold. Number of cases/controls in each group is indicated on the left y-axis and the HRs on the right y-axis. (c) Risk of any fracture among T2DM patients compared to matched population controls. Analysis stratified per level of physical activity. The risk of any fracture was analyzed using multivariable-adjusted Cox models. A 20% risk increase (HR 1.20) was used as threshold for risk increase. Number of cases/controls in each group is indicated on the left y-axis and the HRs on the right y-axis. (d) Risk of any fracture among T2DM patients with or without insulin treatment last year compared to matched population controls. The risk of any fracture was analyzed using multivariable-adjusted Cox models. A 20% risk increase (HR 1.20) was used as threshold for risk increase. Number of cases/controls in each group is indicated on the left y-axis and the HR on the right y-axis.
(DOCX)

S9 Fig Risk of fracture in T2DM patients compared to matched controls—per age and number of risk factors. (DOCX)

S1 Table Detailed definitions of outcomes. Fracture data was refined in multiple steps. First, fracture diagnoses with a simultaneous code indicating a revisit (Z09, Z47, Z48) and hip fracture diagnoses without a simultaneous code for surgical procedure were discarded. Second, a washout period of 5 months was used, so that if a fracture diagnosis referring to the same skeletal site was repeated within a period of 5 months, the latter diagnosis was discarded to avoid including codes from revisits. Incident hip fracture included fractures of the femoral head, neck, trochanter, or subtrochanteric part of the femur accompanied with a code for surgical procedure (NFB, NFC, or NFJ).
(DOCX)

S2 Table Detailed definitions of covariates. (DOCX)

S3 Table Outcomes for T2DM patients vs. controls according to sex. Subgroup analyses per sex. Outcomes for patients with type 2 diabetes and population controls without diabetes, matched according to birth year, sex, and county. Event rates were calculated as the number of persons with respective outcome per 1,000 person-years and are presented with exact Poisson 95% CIs. The adjusted Cox model is adjusted for age, sex, sickness benefits, marital status, urban residency, non-Nordic citizenship at birth, Charlson comorbidity index, osteoporosis diagnosis, conditions associated with osteoporosis, alcohol-related disease, rheumatoid arthritis, osteoporosis medication, calcium + vitamin D, oral prednisolone, prevalent fracture, prevalent fall injury, nitrates, diuretics, thiazides, beta blockers, calcium channel blockers, RAS inhibitors, and statins.
(DOCX)

S4 Table Baseline characteristics of patients with type 2 diabetes and controls according to sex. (DOCX)

S5 Table Other outcomes for patients with type 2 diabetes and controls. Outcomes for patients with type 2 diabetes and population controls without diabetes, matched according to birth year, sex, and county. Event rates were calculated as the number of persons with respective outcome per 1,000 person-years and are presented with exact Poisson 95% CIs. The adjusted Cox model is adjusted for age, sex, sickness benefits, marital status, urban residency, non-Nordic citizenship at birth, Charlson comorbidity index, osteoporosis diagnosis, conditions associated with osteoporosis, alcohol-related disease, rheumatoid arthritis, osteoporosis medication, calcium + vitamin D use, oral prednisolone medication use, prevalent fracture, prevalent fall injury, nitrates, diuretics, thiazides, beta blockers, calcium channel blockers, renin-angiotensin system inhibitors, and statins.
(DOCX)

S6 Table Subdistribution hazard ratios for T2DM patients vs. population controls with consideration of competing risk of death. Subdistribution hazard ratios with 95% CI for T2DM vs. controls for fractures and injurious falls. Calculated in a subset of 50.000 randomly selected persons using a Fine and Grey model with death as the competing risk.
(DOCX)

## Abbreviations

BMD: bone mineral density
CI: confidence interval
HR: hazard ratio
IQR: interquartile range
MOF: major osteoporotic fracture
SD: standard deviation
SMD: standardized mean difference
T2DM: type 2 diabetes mellitus
