# Supplementary material for: Risk of fracture in adults with type 2 diabetes in Sweden: A national cohort study
Source: PLoS Med. 2023 Jan 26;20(1):e1004172. doi: 10.1371/journal.pmed.1004172 (PMC9910793; doi:10.1371/journal.pmed.1004172)
Supplement: S1 Appendix — (DOCX) [file pmed.1004172.s001.docx]

### S1 Appendix STROBE Statement - Checklist of items that should be included in reports of cohort studies

|  | **Item No** | **Recommendation** |
| --- | --- | --- |
| **Title and abstract** | 1 | (*a*) Indicate the study’s design with a commonly used term in the title or the abstract |
|  |  | *"Cohort study" stated in both the title and the abstract* |
|  |  | (*b*) Provide in the abstract an informative and balanced summary of what was done and what was found |
|  |  | *Written as recommended. Also, a special author summary included.* |
| **Introduction** |  |  |
| Background/rationale | 2 | Explain the scientific background and rationale for the investigation being reported |
|  |  | *Explained in the introduction.* |
| Objectives | 3 | State specific objectives, including any prespecified hypotheses |
|  |  | *This has been stated in the last paragraph of the introduction.* |
| **Methods** |  |  |
| Study design | 4 | Present key elements of study design early in the paper |
|  |  | *This is presented in methods, paragraph “Study design”, first sentence* |
| Setting | 5 | Describe the setting, locations, and relevant dates, including periods of recruitment, exposure, follow-up, and data collection |
|  |  | *This has been described in the paragraph “Study Design and Data Sources”.* |
| Participants | 6 | (*a*) Give the eligibility criteria, and the sources and methods of selection of participants. Describe methods of follow-up |
|  |  | *Criteria for eligible patients are stated in methods, second paragraph of “Study Design and Data Sources”* |
|  |  | *Follow-up is described in the method, second paragraph in "Statistics".* |
|  |  | (*b*) For matched studies, give matching criteria and number of exposed and unexposed |
|  |  | *The matching criteria are presented in methods under in the last paragraph of “Study Design and Data Sources”.* |
|  |  | *The number of exposed and unexposed are presented in the first sentence of the results.* |
| Variables | 7 | Clearly define all outcomes, exposures, predictors, potential confounders, and effect modifiers. Give diagnostic criteria, if applicable |
|  |  | *Defined in methods under the heading “Outcomes”* |
| Data sources/ measurement | 8* | For each variable of interest, give sources of data and details of methods of assessment (measurement). Describe comparability of assessment methods if there is more than one group |
|  |  | *Data sources are described in the methods section under the paragraph “Study Design and Data Sources”.* |

| Bias | 9 | Describe any efforts to address potential sources of bias |
| --- | --- | --- |
|  |  | *Described in methods in under the paragraph “Statistical analyses”. In particular,* |
|  |  | *(i) controls were selected using replacement in order to avoid replacement bias* |
|  |  | *(ii) adjustments were made to account for differences between the T2DM patients and the controls* |
|  |  | *(iii) yearly incidence rates were calculated to explore possible temporal biases* |
| Study size | 10 | Explain how the study size was arrived at |
|  |  | *The study size is a consequence of time period chosen (January 1^st^ 2007 -December 31^st^ 2017) and the diagnostic inclusion and exclusion criteria as explained in methods under the paragraph “Study Design and Data Sources”* |
| Quantitative variables | 11 | Explain how quantitative variables were handled in the analyses. If applicable, describe which groupings were chosen and why |
|  |  | *Continuous variables: Age, BMI, blood pressure, HbA1c, Cholesterol, age at diagnosis and medium duration of diabetes at baseline* |
|  |  | *Ordinal variables: The Charlson Comorbidity (though presented in Table 1 in groups of 0, 1 or 2 or ≥3 for brevity). When adjusting for Charlson comorbidity index, it was made using it as an ordinal variable.* |
|  |  | *Categorical variables: Marital status (per definition), Physical activity (per definition), Chronic Kidney Disease (CKD) stage was derived based on the GFR value. The categorical CKD variable was used when adjusting.* |
| Statistical methods | 12 | (*a*) Describe all statistical methods, including those used to control for confounding |
|  |  | *Described in methods under “Statistical analyses”* |
|  |  | (*b*) Describe any methods used to examine subgroups and interactions |
|  |  | *Described in methods under “Statistical analyses”* |
|  |  |  |
|  |  | (*c*) Explain how missing data were addressed |
|  |  |  |
|  |  | *The main analyses used electronic health records with high validity regarding diagnoses and prescribed medications, thus missing data was interpreted as non-existing.* |
|  |  | *Regarding the data originating from the National Diabetes Register, not all variables were available for all patients. Thus these were imputed and analyses with complete cases were made as sensitivity analyses* |
|  |  | *Regarding fracture diagnoses, we excluded fracture diagnosis if repeated on the same skeletal site (revisit and not a new fracture), using a validated procedure described under “Variable definitions”.* |

|  |  | (*d*) If applicable, explain how loss to follow-up was addressed |
| --- | --- | --- |
|  |  |  |
|  |  | *We censored the Cox model for death, emigration and end of study. The controls were also censored for diabetes, i.e. NDR registration, diagnosis or prescription in order to ensure that the controls were free from T2DM. Described in methods under “Statistical analyses”, second paragraph, row 4-5.* |
|  |  | (*e*) Describe any sensitivity analyses |
|  |  | *This is described in detail in “Statistical analyses”.* |
| **Results** |  |  |
| Participants | 13* | (a) Report numbers of individuals at each stage of study—eg numbers potentially eligible, examined for eligibility, confirmed eligible, included in the study, completing follow-up, and analysed |
|  |  | (b) Give reasons for non-participation at each stage |
|  |  | (c) Consider use of a flow diagram |
|  |  | *We describe patients included in the study in methods, second paragraph of "Study Design and Data Sources"* |
| Descriptive data | 14* | (a) Give characteristics of study participants (eg demographic, clinical, social) and information on exposures and potential confounders |
|  |  | *This data is presented in Table 1.* |
|  |  | (b) Indicate number of participants with missing data for each variable of interest |
|  |  | *This data is presented in Table 1, part B* |
|  |  | (c) Summarise follow-up time (eg, average and total amount) |
|  |  | *This is stated in the abstract as well as in the end of the first paragraph of results.* |
| Outcome data | 15* | Report numbers of outcome events or summary measures over time |
|  |  | *This is reported in the second and forth paragraph of result and referenced to Table 2 and Figure 1* |
| Main results | 16 | (*a*) Give unadjusted estimates and, if applicable, confounder-adjusted estimates and their precision (eg, 95% confidence interval). Make clear which confounders were adjusted for and why they were included |
|  |  | *Unadjusted hazard ratios are presented in the second and fourth paragraph of the result and together with incident rates per 1000 person-years and adjusted hazard rations in Table 2* |
|  |  | (*b*) Report category boundaries when continuous variables were categorized |
|  |  | Not applicable |
|  |  | (*c*) If relevant, consider translating estimates of relative risk into absolute risk for a meaningful time period |
|  |  | *Five-year absolute risk differences are presented in Figure 3 depending on number of risk factors.* |

| Other analyses | 17 | Report other analyses done—eg analyses of subgroups and interactions, and sensitivity analyses |
| --- | --- | --- |
|  |  | *In the results and Supplemental Figure S3, the association between T2DM and any fracture are presented per sex, age group, prevalent fracture, Charlson comorbidity Index. Also analyses to investigate the potential impact of competing risk are included.* |
| **Discussion** |  |  |
| Key results | 18 | Summarise key results with reference to study objectives |
|  |  | *First paragraph in Discussion.* |
| Limitations | 19 | Discuss limitations of the study, taking into account sources of potential bias or imprecision. Discuss both direction and magnitude of any potential bias |
|  |  | *We discuss the limitations of the study.* |
| Interpretation | 20 | Give a cautious overall interpretation of results considering objectives, limitations, multiplicity of analyses, results from similar studies, and other relevant evidence |
|  |  | *A cautious interpretation is stated in the last concluding paragraph of the discussion.* |
| Generalisability | 21 | Discuss the generalisability (external validity) of the study results |
|  |  | *The fact that national studies not necessarily is applicable to other country settings is implied.* |
| **Other information** |  |  |
| Funding | 22 | Give the source of funding and the role of the funders for the present study and, if applicable, for the original study on which the present article is based |
|  |  | *The funding sources and their role are described in the first paragraph of the methods* |
|  |  |  |
|  |  |  |
| *Give information separately for exposed and unexposed groups. | | |
