# Supplementary material for: Risk of fracture in adults with type 2 diabetes in Sweden: A national cohort study
Source: PLoS Med. 2023 Jan 26;20(1):e1004172. doi: 10.1371/journal.pmed.1004172 (PMC9910793; doi:10.1371/journal.pmed.1004172)
Supplement: S2 Appendix — Additional information on registers used. Standardized differences. (DOCX) [file pmed.1004172.s002.docx]

**S2 Appendix Extended Methods**

### Additional Information on Registers used

National Patient Register: Started in 1964 with diagnoses from admitted patients which became compulsory nationally in 1987. Diagnoses from outpatient visits were added in 2001. Only diagnoses set by physicians are included. We only used ICD-10 codes, implemented which has been registered since 1998.

Swedish Prescribed Drug Register: Started July 1st 2005 and includes ATC-codes of prescribed and collected medications.

### Standardized Mean Differences (SMD)

For categorical variables, standardized mean differences were calculated as follows:

<https://support.sas.com/resources/papers/proceedings12/335-2012.pdf>

Otherwise, standardized mean differences were calculated as follows:

SMD = |μ_cases_ - μ_controls_| / [(σ_cases_^2^ + σ_controls_^2^) / 2]
