# Supplementary material for: Risk of fracture in adults with type 2 diabetes in Sweden: A national cohort study
Source: PLoS Med. 2023 Jan 26;20(1):e1004172. doi: 10.1371/journal.pmed.1004172 (PMC9910793; doi:10.1371/journal.pmed.1004172)
Supplement: S1 Fig — Variables from the Diabetes Register with 60% registration rate or higher were included and imputed using the MICE-package in R-Studio (Multivariate Imputation by Chained Equations), using 20 iterations with Nelson–Aalen estimates for all the outcomes. In addition to the outcomes, all the variables included in Table 1 were included in the imputation. Histograms without and with imputation are presented below for the following variables: (a) BMI (kg/m2). (b) Systolic blood pressure (mmHg). (c) Diastolic blood pressure (mmHg). (d) HbA1c (mmole/mole). (e) Cholesterol (mmole/liter). (f) Duration of diabetes (years). (g) Smoker (yes/no) Smoker = 1 cigarette per day or more or pipe smoker. Includes those who stopped smoking less than 3 months earlier. (h) Physical activity (30 min walk or equivalent, times per week). Groups of physical activity (30 minutes’ walk or equivalent) 1: Never, 2: <1 per week, 3: 1–2 per week, 4: 3–5 per week, 5: Daily. (i) Chronic kidney disease (renal failure), groups: No (GFR ≥60), 1: Moderate (GFR 30–59.9), 2: Severe (GFR 15–29.9), 3: Terminal (GFR <15)). (DOCX) [file pmed.1004172.s003.docx]

## S1 Figure a-i: Histograms Without and With Imputation

Variables from the Diabetes Register with 60% registration rate or higher were included and imputed using the MICE-package in R-Studio (Multivariate Imputation by Chained Equations), using twenty iterations with Nelson-Aalen estimates for all the outcomes. In addition to the outcomes, all the variables included in Table 1 were included in the imputation.

Histograms without and with imputation are presented below for the following variables:

1. BMI (kg/m^2^)
2. Systolic blood pressure (mmHg)
3. Diastolic blood pressure (mmHg)
4. HbA1c (mmole/mole)
5. Cholesterol (mmole/liter)
6. Duration of diabetes (years)
7. Smoker (yes/no)
8. Physical activity (30 min walk or equivalent, times per week)
9. Chronic Kidney Disease (No (GFR ≥60), 1:Moderate (GFR 30-59.9), 2: Severe (GFR 15-29.9), 3: Terminal (GFR <15))


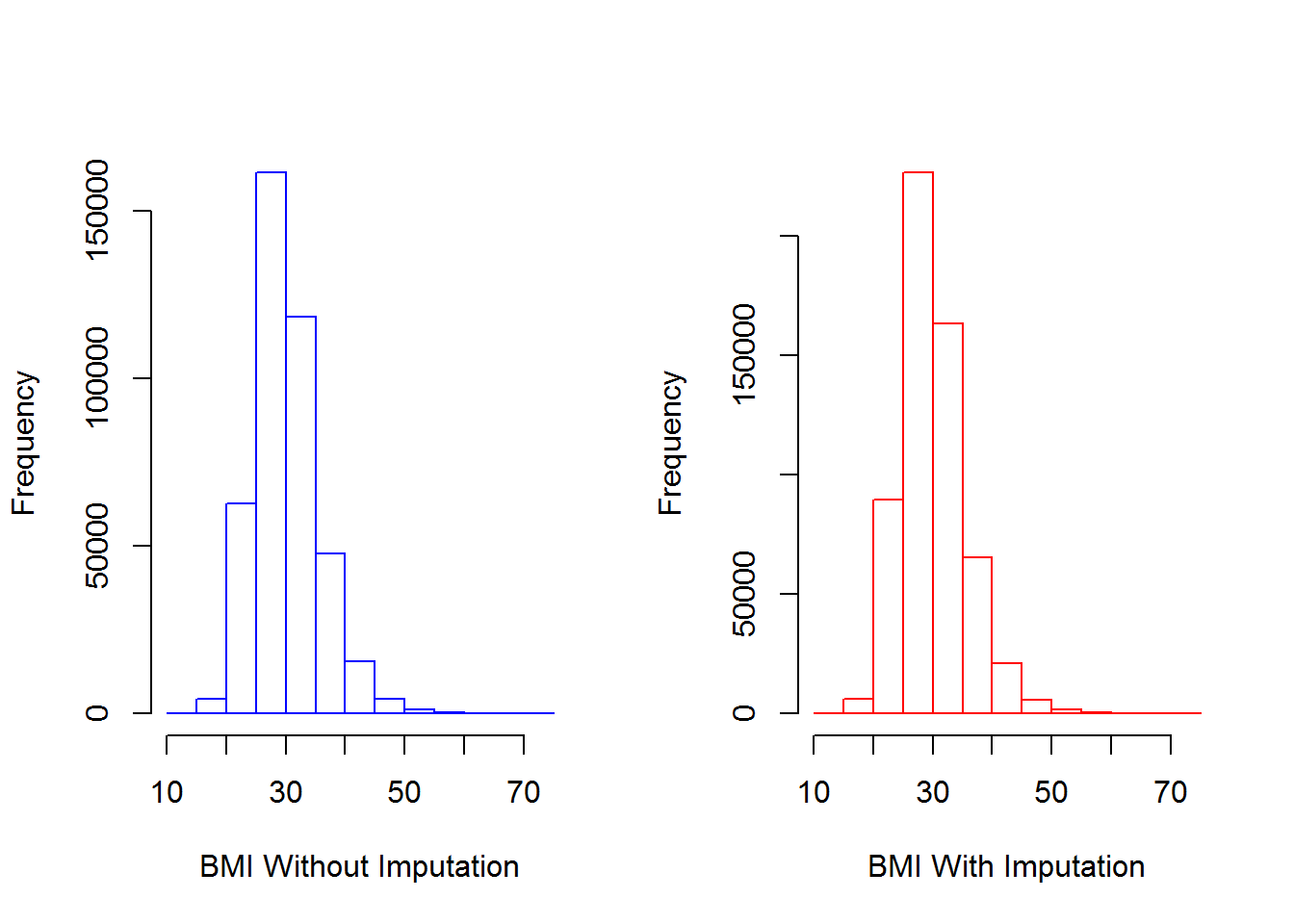


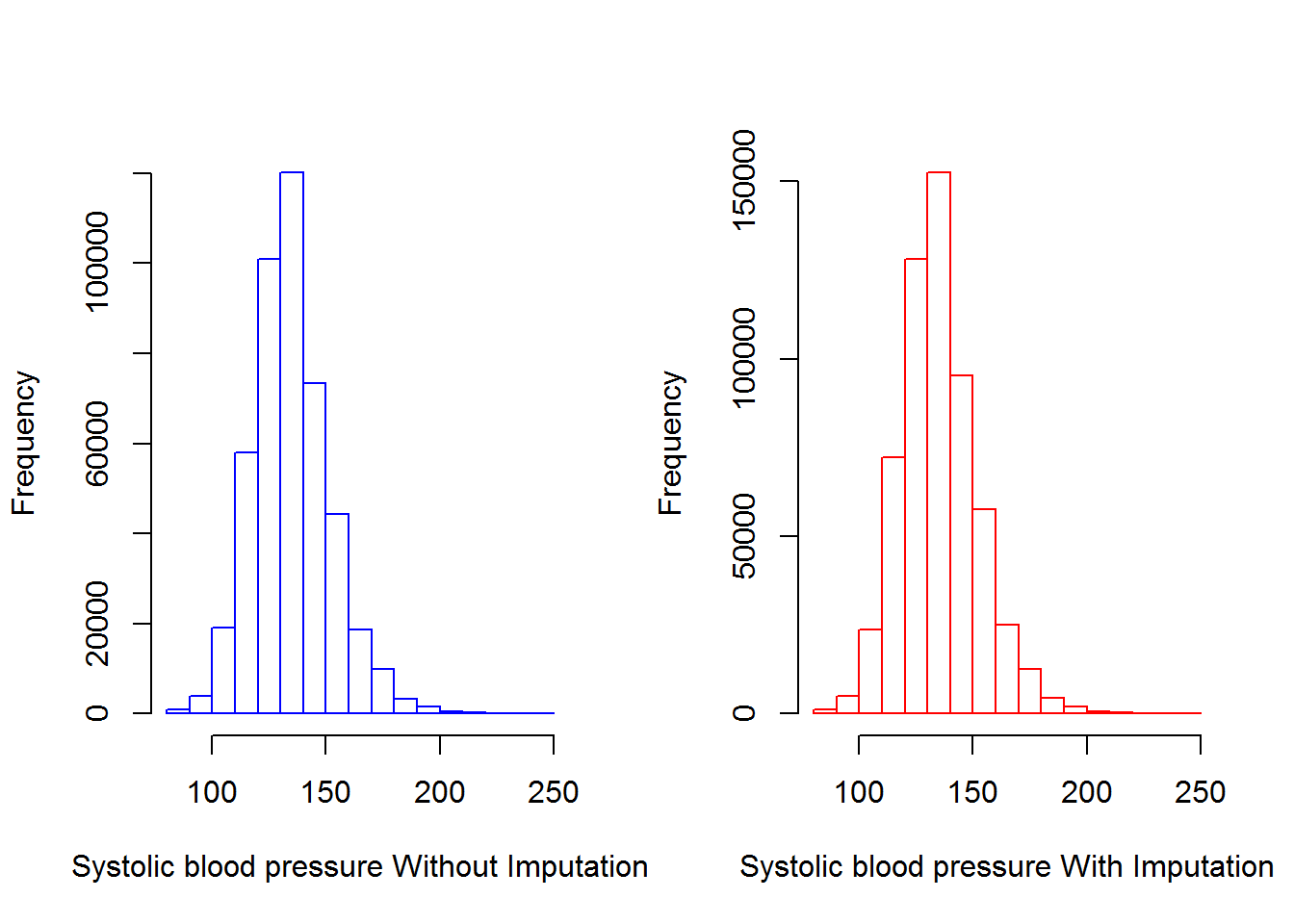


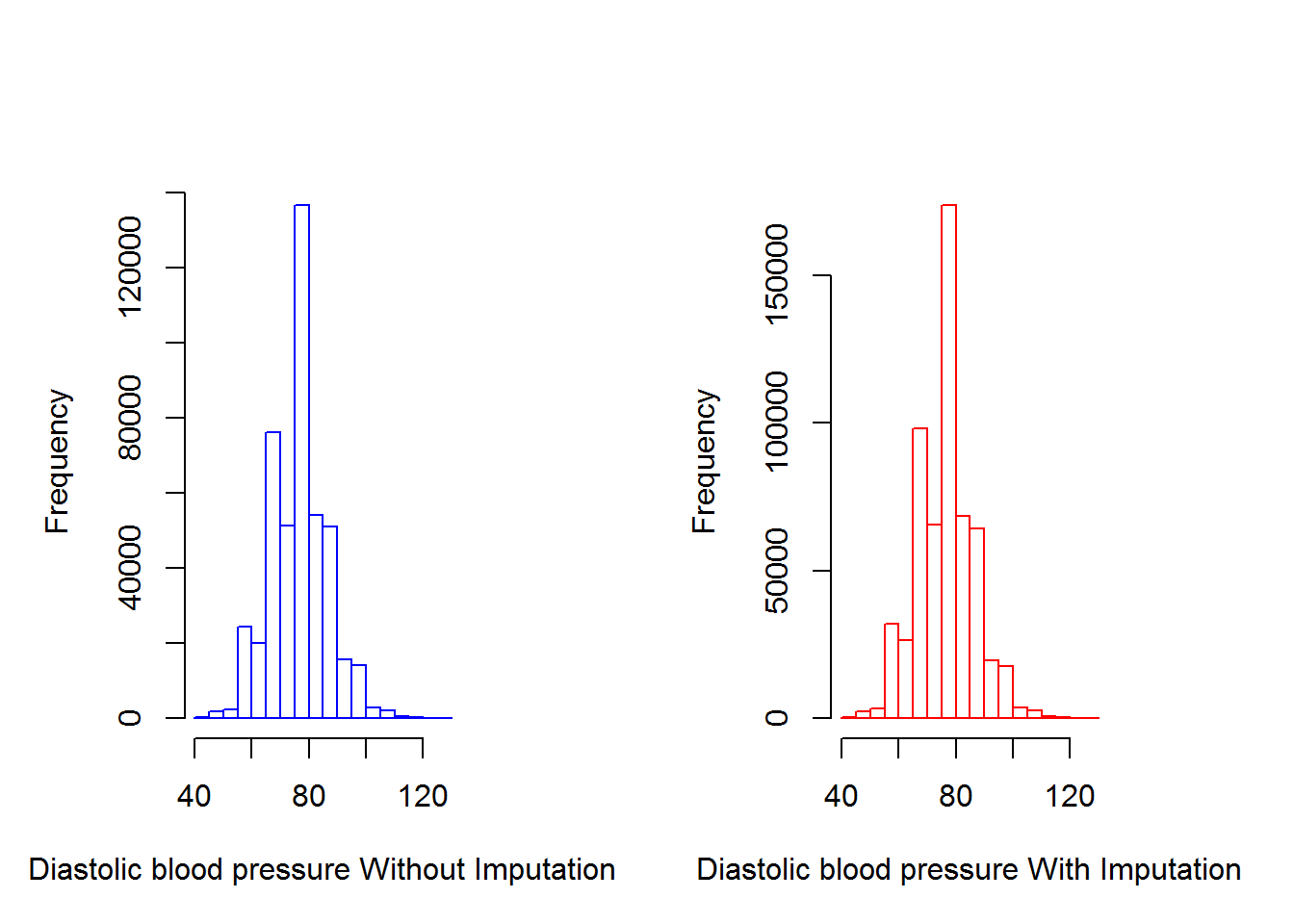


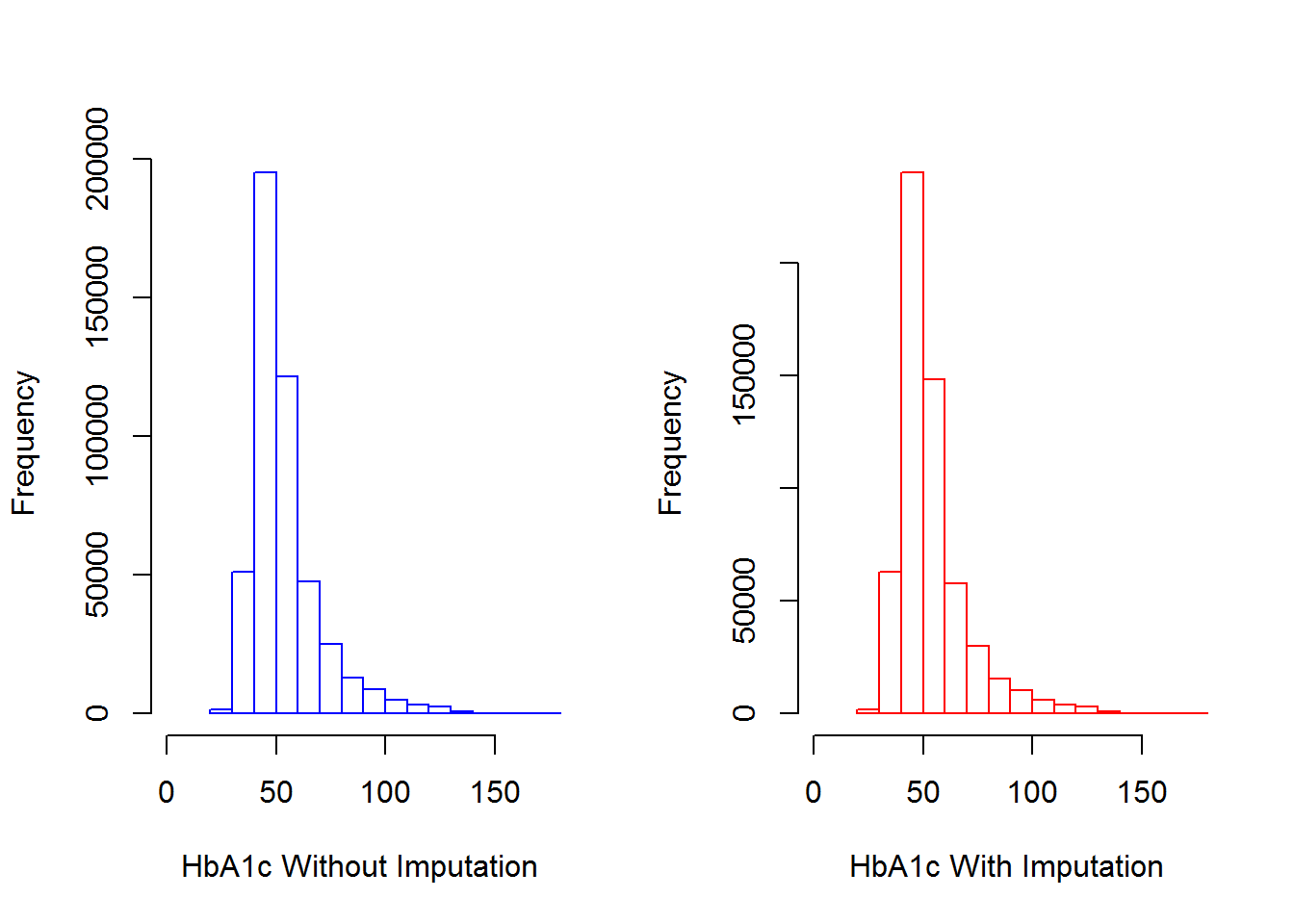

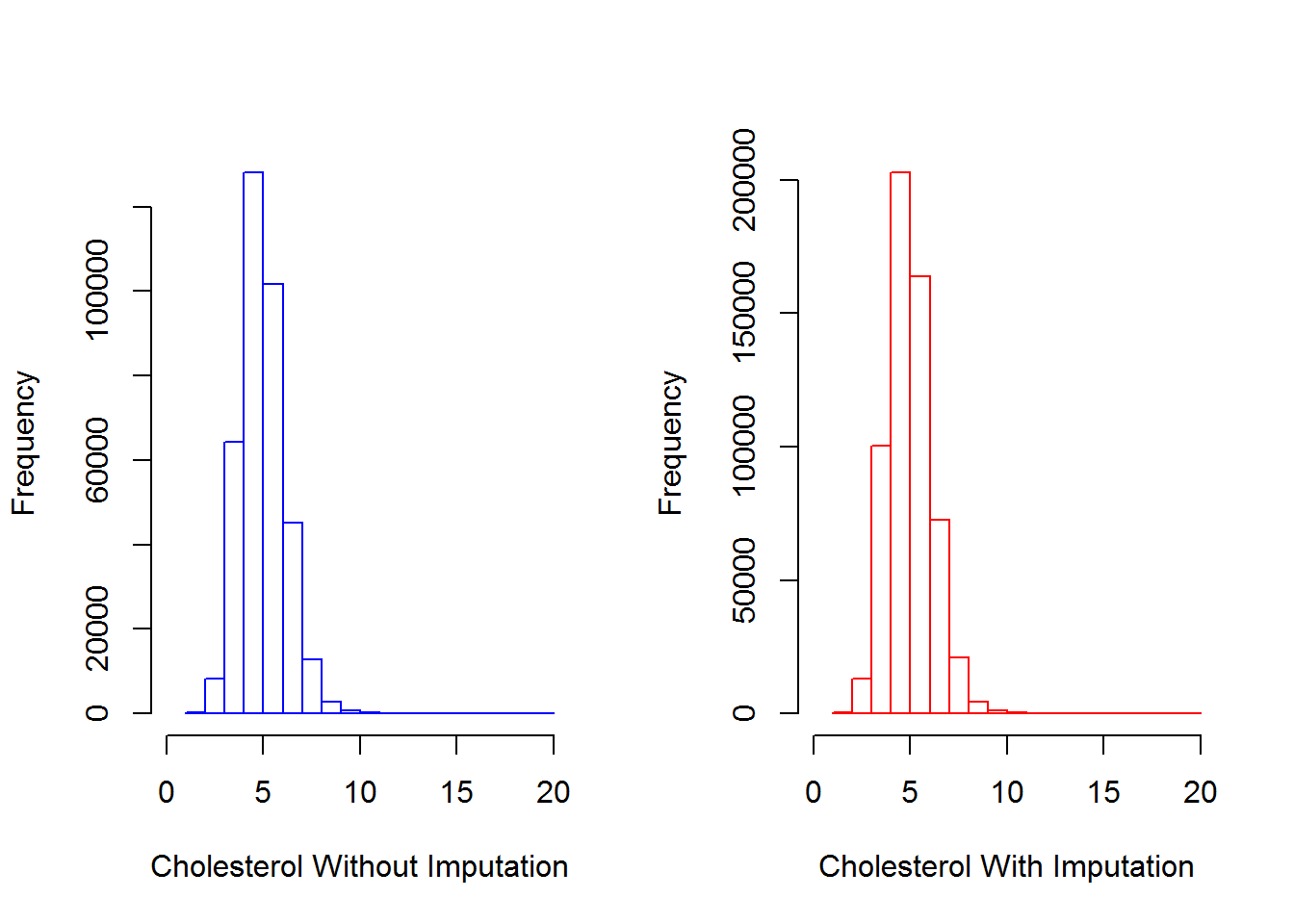


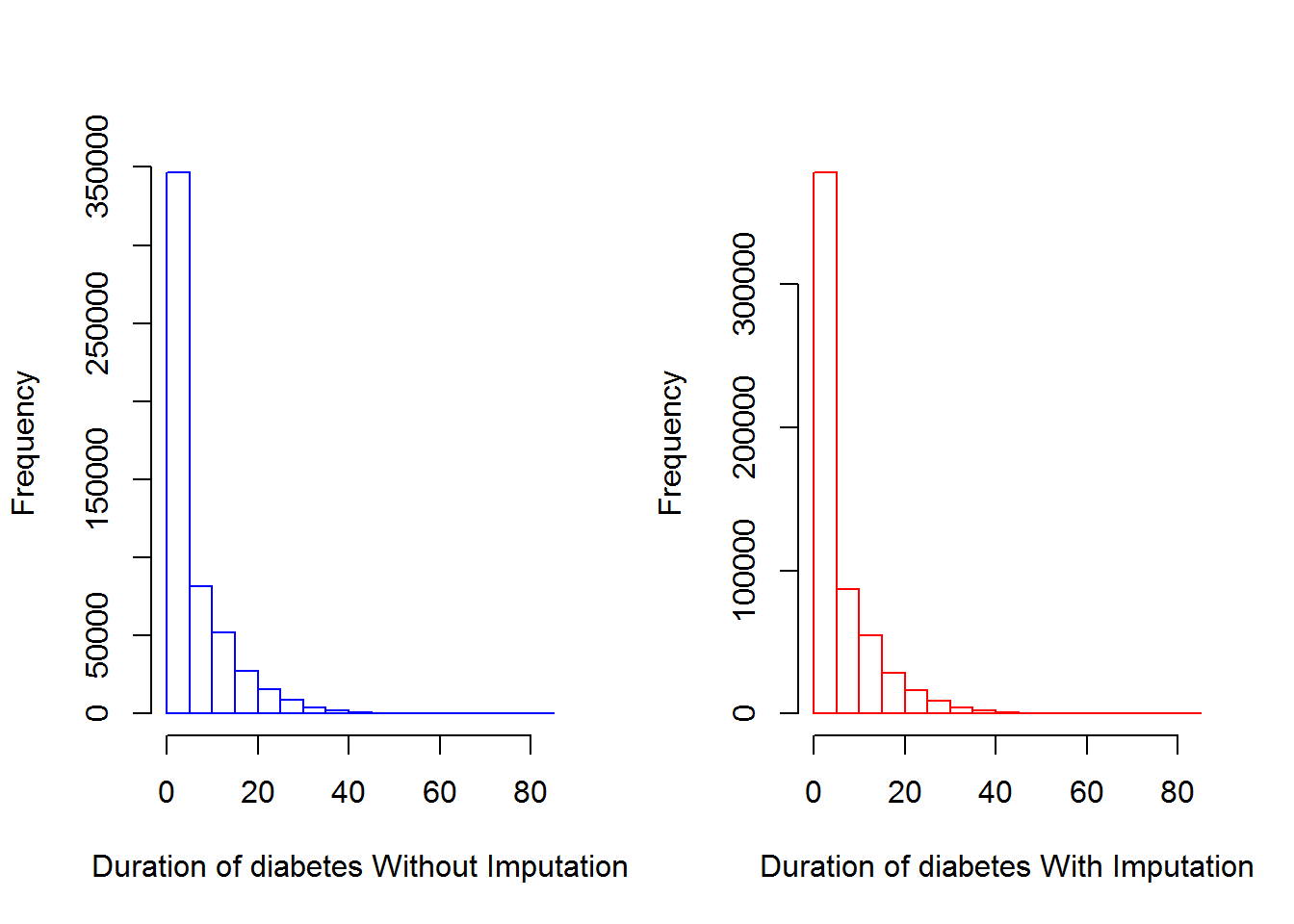


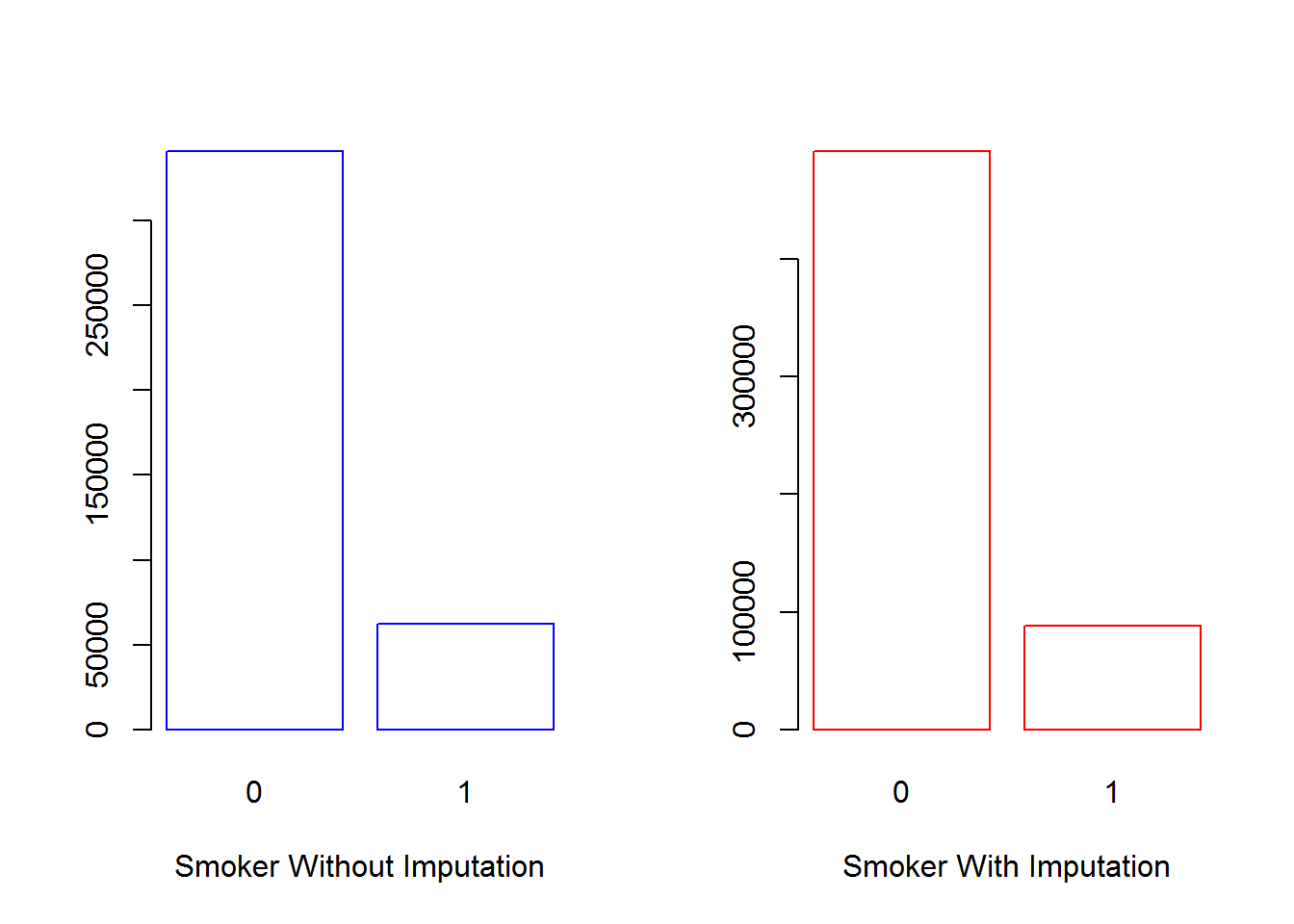


Smoker=1 cigarette per day or more or pipe smoker. Includes those who stopped smoking less than three months earlier


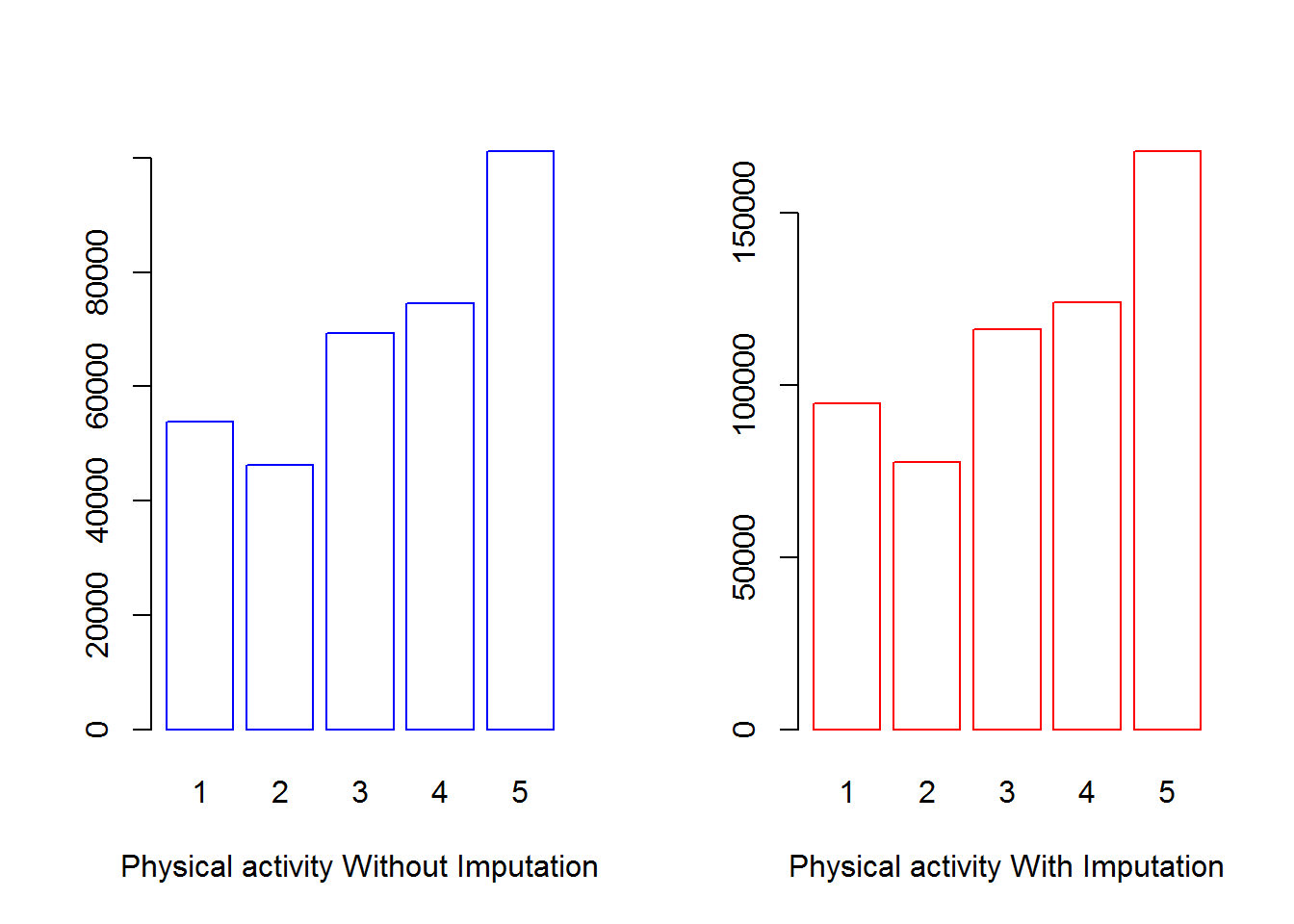


Groups of physical activity (30 minutes’ walk or equivalent)

1: Never, 2: <1 per week, 3: 1-2 per week, 4: 3-5 per week, 5: Daily


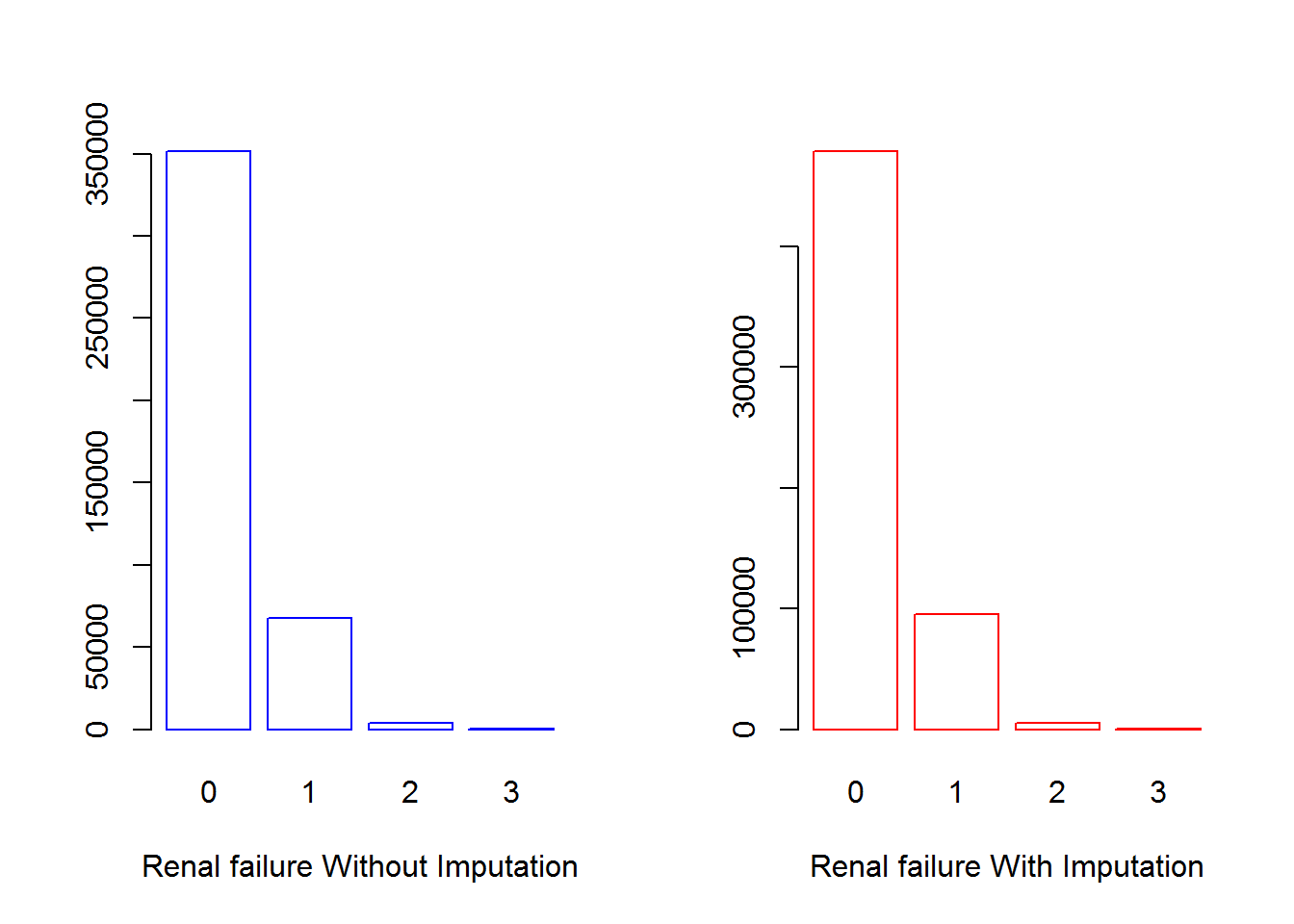


Groups of Chronic Kidney Disease (Renal failure):
0: No (GFR ≥60), 1:Moderate (GFR 30-59.9), 2: Severe (GFR 15-29.9), 3: Terminal (GFR <15)
