# Supplementary material for: Risk of fracture in adults with type 2 diabetes in Sweden: A national cohort study
Source: PLoS Med. 2023 Jan 26;20(1):e1004172. doi: 10.1371/journal.pmed.1004172 (PMC9910793; doi:10.1371/journal.pmed.1004172)
Supplement: S2 Fig — Yearly event rates were estimated as the number of events occurring during each year divided by the number of person-years accumulated during each year, standardized to the age and sex distribution in the entire cohort and presented as event rates per 1,000 person years with 95% CIs based on a normal approximation accounting for the weights. (DOCX) [file pmed.1004172.s004.docx]

## S2 Figure: Yearly Incidence Rates in T2DM Patients vs. Population Controls


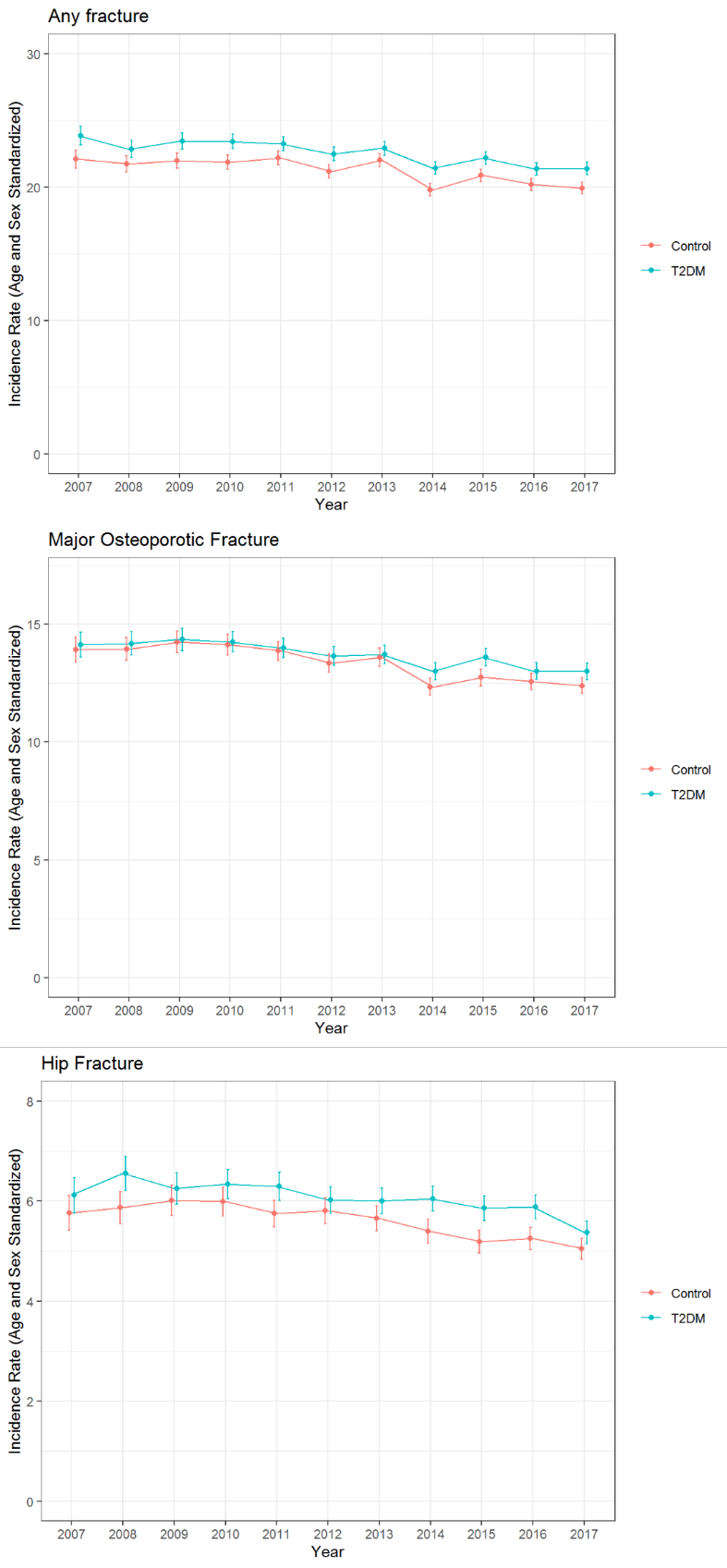


**Yearly Incidence Rates in T2DM Patients vs. Population Controls.**

Yearly incident rates were estimated as the number of events occurring during each year divided by the number of person-years accumulated during each year, standardized to the age and sex distribution in the entire cohort and presented as event rates per 1000 person years with 95% confidence intervals based on a normal approximation accounting for the weights.
