# Supplementary material for: Risk of fracture in adults with type 2 diabetes in Sweden: A national cohort study
Source: PLoS Med. 2023 Jan 26;20(1):e1004172. doi: 10.1371/journal.pmed.1004172 (PMC9910793; doi:10.1371/journal.pmed.1004172)
Supplement: S3 Fig — P-values for the interaction term are stated per group. HRs for any fracture were calculated in Cox models adjusted for age, sex, sickness benefits, marital status, urban residency, non-Nordic citizenship at birth, Charlson comorbidity index, osteoporosis diagnosis, conditions associated with osteoporosis, alcohol-related disease, rheumatoid arthritis, osteoporosis medication, calcium + vitamin D use, oral prednisolone medication use, prevalent fracture, prevalent fall injury, nitrates, diuretics, thiazides, beta blockers, calcium channel blockers, renin-angiotensin system inhibitors, and statins. (DOCX) [file pmed.1004172.s005.docx]

## S3 Figure: Multivariable Adjusted Risk of Any Fracture in T2DM Patients vs. Controls – Subgroup Analyses per Age, Sex, Previous Fracture and Charlson Comorbidity Index


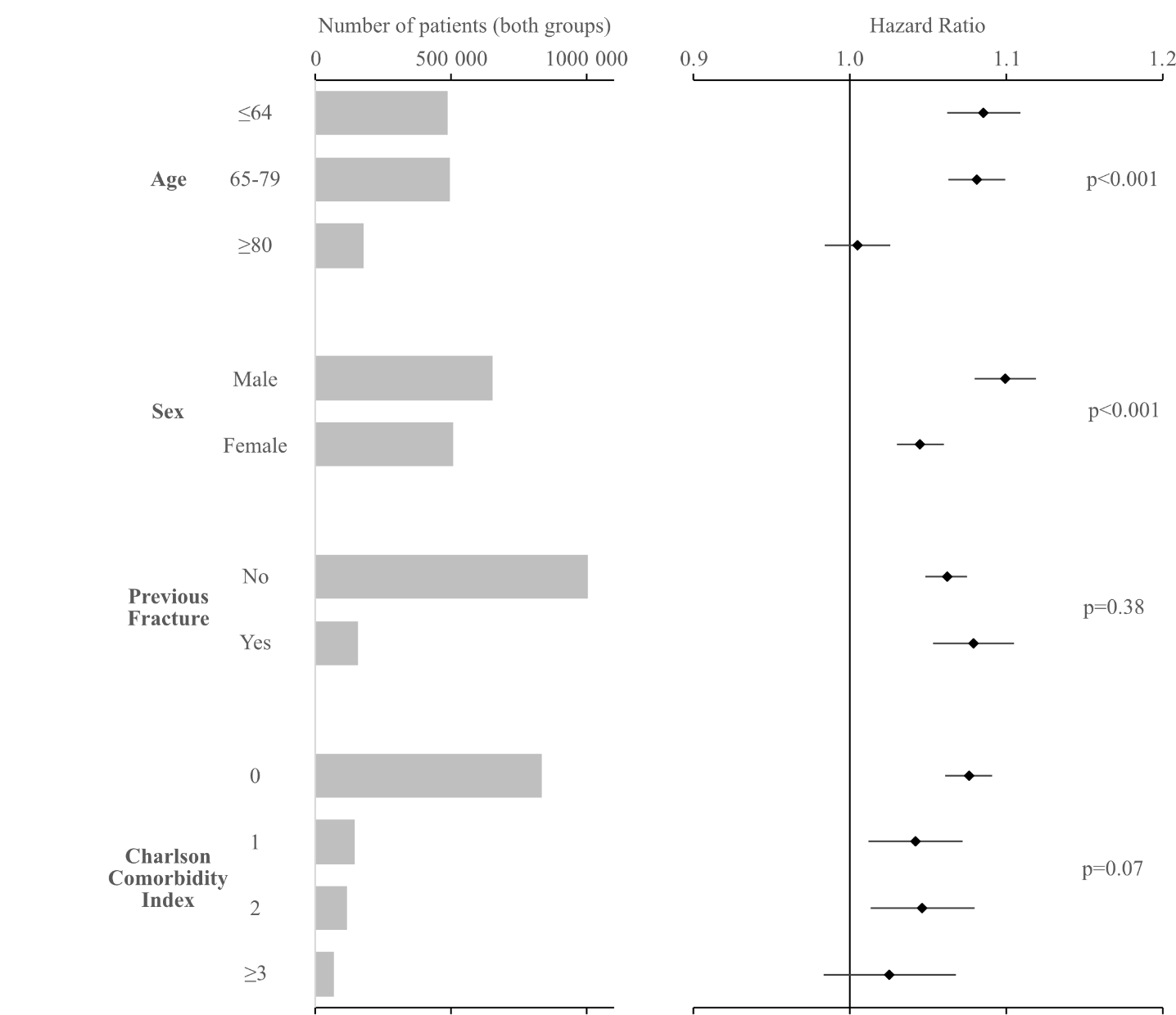


P-values for the interaction term is stated per group. Hazard Ratios for any fracture were calculated in Cox models adjusted for age, gender, sickness benefits, marital status, urban residency, non-Nordic citizenship at birth, Charlson comorbidity index, osteoporosis diagnosis, conditions associated with osteoporosis, alcohol related disease, rheumatoid arthritis, osteoporosis medication, calcium + vitamin D use, oral prednisolone medication use, prevalent fracture, prevalent fall injury, nitrates, diuretics, thiazides, beta blockers, calcium channel blockers, renin-angiotensin system (RAS) inhibitors and statins.
