# Supplementary material for: Risk of fracture in adults with type 2 diabetes in Sweden: A national cohort study
Source: PLoS Med. 2023 Jan 26;20(1):e1004172. doi: 10.1371/journal.pmed.1004172 (PMC9910793; doi:10.1371/journal.pmed.1004172)
Supplement: S4 Fig — The cumulative incidence function, or subdistribution function, of fracture/injurious fall with death as competing risk was estimated using the Aalen–Johansen estimator. All patients included. (DOCX) [file pmed.1004172.s006.docx]

## S4 Figure: Cumulative Incidence Function in T2DM Patients vs. Population Controls


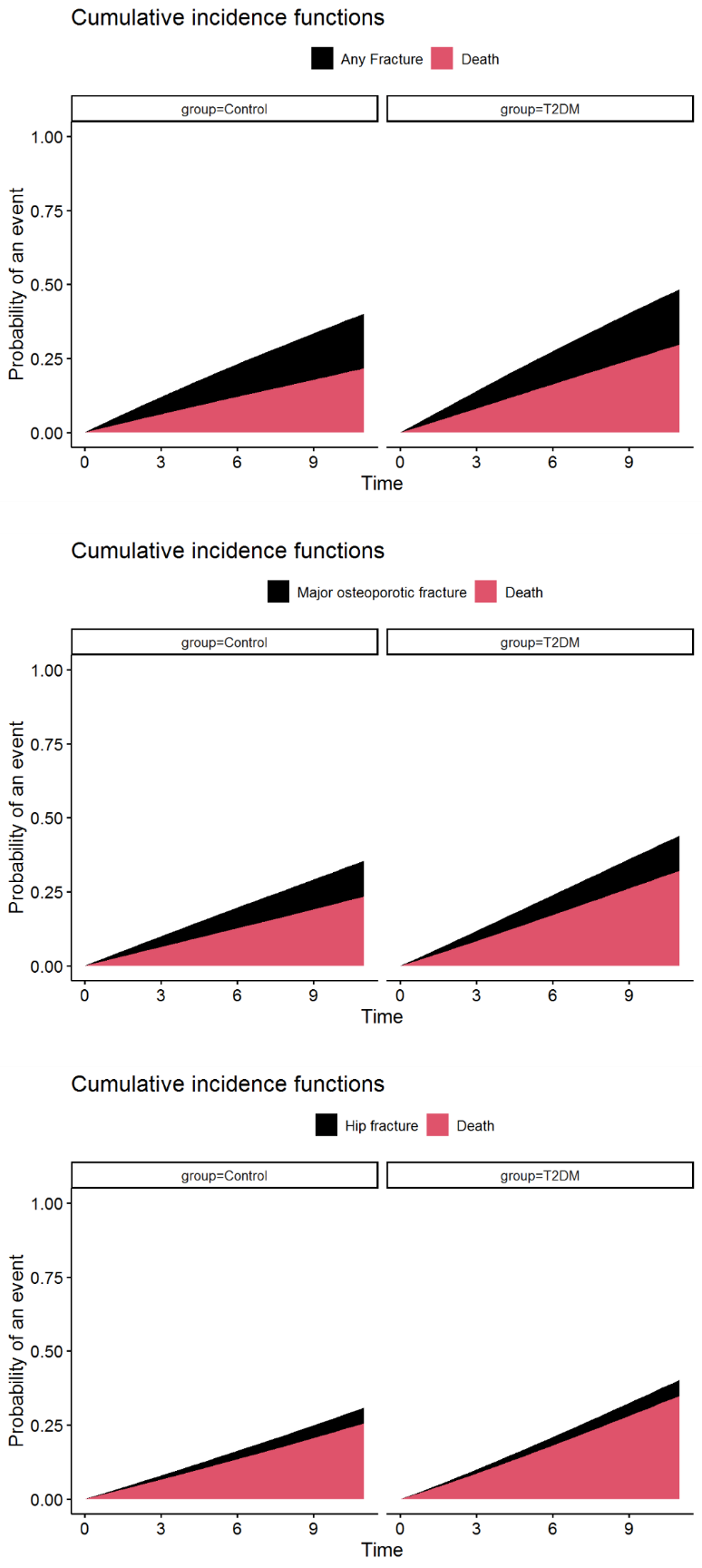


**Cumulative Incidence Function in T2DM Patients vs. Population Controls.** The cumulative incidence function, or subdistribution function, of fracture/injurious fall with death as competing risk was estimated using the Aalen-Johansen estimator. All patients included.
