# Supplementary material for: Risk of fracture in adults with type 2 diabetes in Sweden: A national cohort study
Source: PLoS Med. 2023 Jan 26;20(1):e1004172. doi: 10.1371/journal.pmed.1004172 (PMC9910793; doi:10.1371/journal.pmed.1004172)
Supplement: S5 Fig — Machine learning using Gradient Boosting Machines was applied to all T2DM cases (N = 580,127, no controls). The settings allowed interaction depth 2, i.e., all pairwise interactions were enabled. All variables in Table 1, both general comorbidity and fracture risk factors as well as specific diabetes-related variables (imputed) were included. For any fracture, the top 4 diabetes-related variables are marked with an asterisk and percentages for relative importance included in the pie chart. The color labels are the same for all 3 outcomes. (DOCX) [file pmed.1004172.s007.docx]

## S5 Fig: Identification of Diabetes Related Risk Factors using Gradient Boosting Machines – all cases including imputed values

**
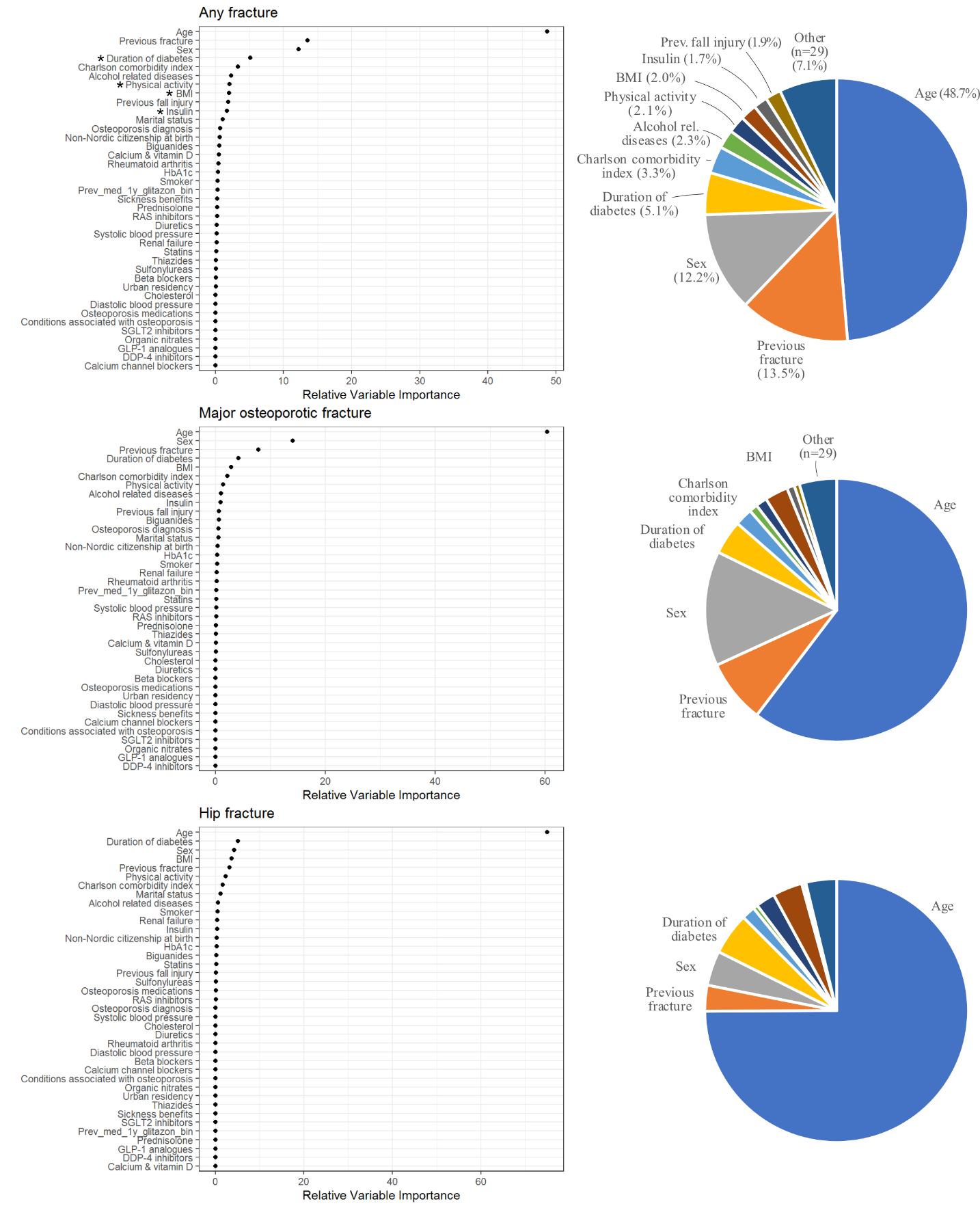
**

Machine learning using Gradient Boosting Machines was applied to all T2DM cases (N=580,127, no controls). The settings allowed interaction depth two, i.e. all pairwise interactions were enabled. All variables in Table 1, both general comorbidity and fracture risk factors as well as specific diabetes related variables (imputed) were included. For any fracture, the top four diabetes related variables are marked with an asterix and percentages for relative importance included in the pie chart. The color labels are the same for all three outcomes.
