# Supplementary material for: Risk of fracture in adults with type 2 diabetes in Sweden: A national cohort study
Source: PLoS Med. 2023 Jan 26;20(1):e1004172. doi: 10.1371/journal.pmed.1004172 (PMC9910793; doi:10.1371/journal.pmed.1004172)
Supplement: S7 Fig — All the T2DM cases (without controls) were included in a Cox regression model, fully adjusted and also including the variables from the Diabetes Register, to investigate the risk of any fracture. Imputed values from the Diabetes Register were included; the continuous variables were splined with 5 degrees of freedom. The figures illustrate the association between the top 4 covariates and any fracture with their respective histograms included. All values are from the same regression model. Numbers of T2DM patients are indicated on the right y-axis and the HRs on the left y-axis. (DOCX) [file pmed.1004172.s009.docx]

**S7 Fig: Analysis of the Top Four Variables Independent Association to Any Fracture**

All the T2DM cases (without controls) were included in a Cox regression model, fully adjusted and also including the variables from the Diabetes Register, to investigate the risk of any fracture. Imputed values from the Diabetes Register were included, the continuous variables were splined with five degrees of freedom. The figures illustrate the association between the top four covariates and any fracture with their respective histograms included. All values are from the same regression model. Number of T2DM patients are indicated on the right y-axis and the Hazard Ratios on the left y-axis.


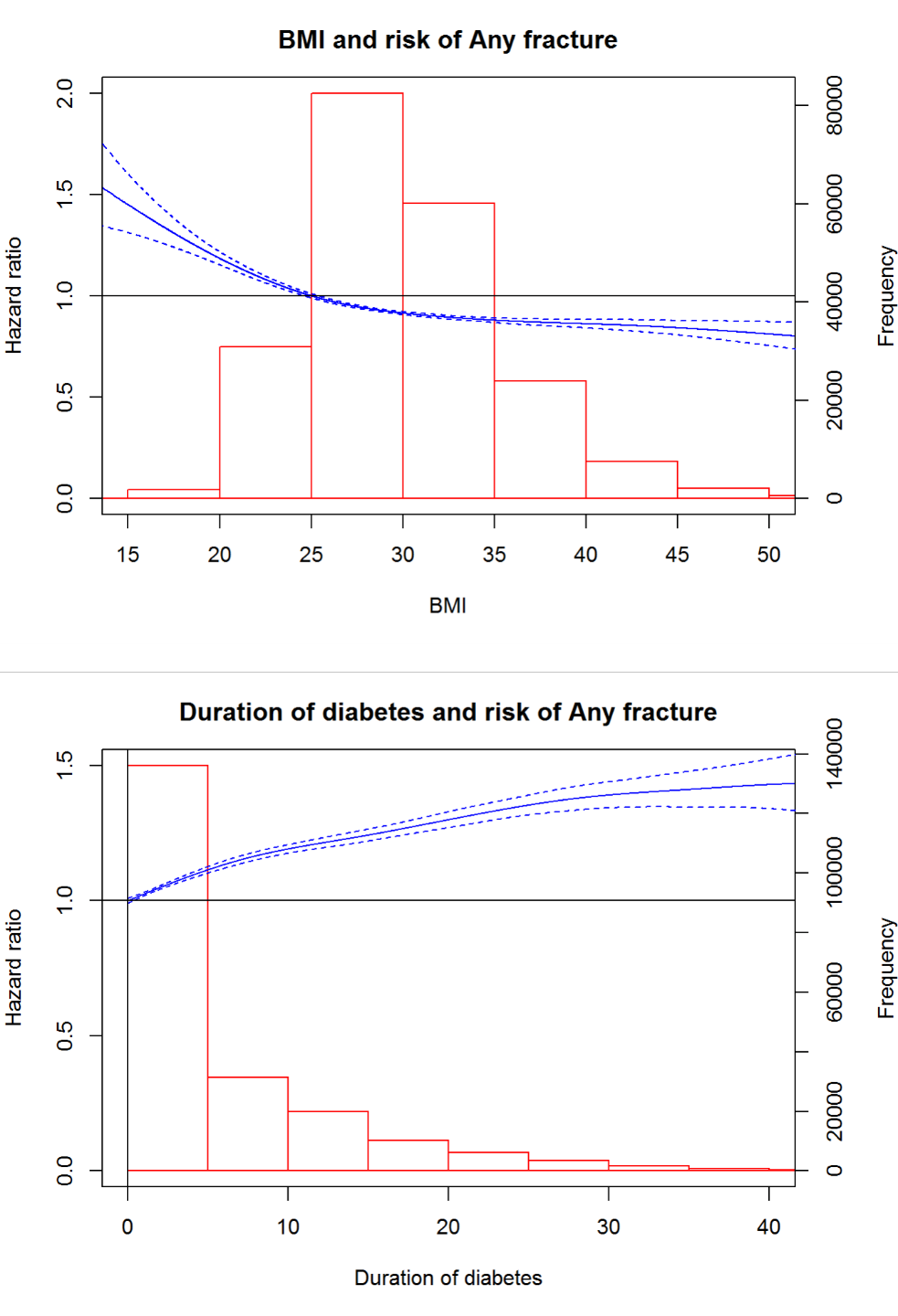

Number of T2DM patients are indicated on the left y-axis and the Hazard Ratios on the right y-axis.

Number of T2DM patients are indicated on the left y-axis and the Hazard Ratios on the right y-axis.
