# Supplementary material for: Risk of fracture in adults with type 2 diabetes in Sweden: A national cohort study
Source: PLoS Med. 2023 Jan 26;20(1):e1004172. doi: 10.1371/journal.pmed.1004172 (PMC9910793; doi:10.1371/journal.pmed.1004172)
Supplement: S8 Fig — (a) Risk of any fracture among T2DM patients compared to matched population controls. The analysis was stratified for BMI. Values of BMI were rounded off to integers. The risk of any fracture was analyzed using multivariable-adjusted Cox models. A 20% risk increase (HR 1.20) was used as a clinically relevant threshold. Number of cases/controls in each group is indicated on the left y-axis and the HRs on the right y-axis. (b) Risk of any fracture among T2DM patients compared to matched population controls. The analysis was stratified for duration of diabetes. Values of duration were rounded off to integers. The risk of any fracture was analyzed using multivariable-adjusted Cox models. A 20% risk increase (HR 1.20) was used as a clinically relevant threshold. Number of cases/controls in each group is indicated on the left y-axis and the HRs on the right y-axis. (c) Risk of any fracture among T2DM patients compared to matched population controls. Analysis stratified per level of physical activity. The risk of any fracture was analyzed using multivariable-adjusted Cox models. A 20% risk increase (HR 1.20) was used as threshold for risk increase. Number of cases/controls in each group is indicated on the left y-axis and the HRs on the right y-axis. (d) Risk of any fracture among T2DM patients with or without insulin treatment last year compared to matched population controls. The risk of any fracture was analyzed using multivariable-adjusted Cox models. A 20% risk increase (HR 1.20) was used as threshold for risk increase. Number of cases/controls in each group is indicated on the left y-axis and the HR on the right y-axis. (DOCX) [file pmed.1004172.s010.docx]

## S8 Figure a-d: Comparison of T2DM Patients to Population Controls – Per Risk Factor

a. Risk of any fracture among T2DM patients compared to matched population controls. The analysis was stratified for BMI. Values of BMI were rounded off to integers. The risk of any fracture was analyzed using multivariable adjusted Cox models. A 20% risk increase (HR 1.20) was used as a clinically relevant threshold. Number of cases/controls in each group is indicated on the left y-axis and the Hazard Ratios on the right y-axis.

b. Risk of any fracture among T2DM patients compared to matched population controls. The analysis was stratified for duration of diabetes. Values of duration were rounded off to integers. The risk of any fracture was analyzed using multivariable adjusted Cox models. A 20% risk increase (HR 1.20) was used as a clinically relevant threshold. Number of cases/controls in each group is indicated on the left y-axis and the Hazard Ratios on the right y-axis.

c. Risk of any fracture among T2DM patients compared to matched population controls. Analysis stratified per level of physical activity. The risk of any fracture was analyzed using multivariable adjusted Cox models. A 20% risk increase (HR 1.20) was used as threshold for risk increase. Number of cases/controls in each group is indicated on the left y-axis and the Hazard Ratios on the right y-axis.

d. Risk of any fracture among T2DM patients with or without insulin treatment last year compared to matched population controls. The risk of any fracture was analyzed using multivariable adjusted Cox models. A 20% risk increase (HR 1.20) was used as threshold for risk increase. Number of cases/controls in each group is indicated on the left y-axis and the Hazard Ratios on the right y-axis.
