# Supplementary material for: Risk of fracture in adults with type 2 diabetes in Sweden: A national cohort study
Source: PLoS Med. 2023 Jan 26;20(1):e1004172. doi: 10.1371/journal.pmed.1004172 (PMC9910793; doi:10.1371/journal.pmed.1004172)
Supplement: S9 Fig — (DOCX) [file pmed.1004172.s011.docx]

## S9 Figure: Risk of Fracture in T2DM Patients Compared to Matched Controls - per Age and Number of Risk Factors
