# Supplementary material for: Risk of fracture in adults with type 2 diabetes in Sweden: A national cohort study
Source: PLoS Med. 2023 Jan 26;20(1):e1004172. doi: 10.1371/journal.pmed.1004172 (PMC9910793; doi:10.1371/journal.pmed.1004172)
Supplement: S1 Table — Fracture data was refined in multiple steps. First, fracture diagnoses with a simultaneous code indicating a revisit (Z09, Z47, Z48) and hip fracture diagnoses without a simultaneous code for surgical procedure were discarded. Second, a washout period of 5 months was used, so that if a fracture diagnosis referring to the same skeletal site was repeated within a period of 5 months, the latter diagnosis was discarded to avoid including codes from revisits. Incident hip fracture included fractures of the femoral head, neck, trochanter, or subtrochanteric part of the femur accompanied with a code for surgical procedure (NFB, NFC, or NFJ). (DOCX) [file pmed.1004172.s012.docx]

## S1 Table: Detailed Definitions of Outcomes

| **Variable** | **Definitions Using ICD-10 Codes** |
| --- | --- |
| Any Fracture | S12, S22, S32, S42, S52, S62 (excluding fingers S625-S627), S72, S82, S92 (excluding toes S924-S925), T02, T08, T10, T12, T142, M485 |
| Major Osteoporotic Fracture | hip (S720-S722), wrist (S525-S526), collum chirugicum (S422), vertebra (S220, S221, S320), pelvis (S32) |
| Hip Fracture | S720-S722 with a code for surgical procedure (NFB, NFC, NFJ) |
| Lower Leg Fracture | S82 |
| Wrist Fracture | S525, S526 |
| Proximal Humerus Fracture | S422 |
| Injurious fall | W00-W19 code and a S00-T14 diagnosis, but not a simultaneous fracture code |

Fracture data was refined in multiple steps. First, fracture diagnoses with a simultaneous code indicating a revisit (Z09, Z47, Z48) and hip fracture diagnoses without a simultaneous code for surgical procedure were discarded. Second, a washout period of five months was used, so that if a fracture diagnosis referring to the same skeletal site was repeated within a period of five months, the latter diagnosis was discarded to avoid including codes from revisits. Incident hip fracture included fractures of the femoral head, neck, trochanter or subtrochanteric part of the femur accompanied with a code for surgical procedure (NFB, NFC or NFJ).
