# Supplementary material for: Risk of fracture in adults with type 2 diabetes in Sweden: A national cohort study
Source: PLoS Med. 2023 Jan 26;20(1):e1004172. doi: 10.1371/journal.pmed.1004172 (PMC9910793; doi:10.1371/journal.pmed.1004172)
Supplement: S2 Table — (DOCX) [file pmed.1004172.s013.docx]

## S2 Table: Detailed Definitions of Covariates

| **Variable** | **Definitions Using ICD-10 Codes and/or ATC-Codes** |
| --- | --- |
| Age | At baseline |
| Sickness benefits | Any payment from the social services during the baseline year |
| Marital status | Categorized as married, unmarried, divorced or widowed. Registered partnerships considered as marriages in respective category. Value taken from end of the baseline year, if missing the adjacent years are used if available. |
| Urban residency, (>200 per km^2^) | The municipal registration at baseline is used and its population density |
| Non-Nordic citizenship at birth | Other citizenships at birth than Swedish, Norwegian, Danish, Finish or Icelandic |
| Charlson comorbidity index (weight) | The weighted sum of the diseases below. Diagnoses from both outpatient visits and admissions used. NOTE: Diabetes not included. |
| Dementia (1) | F00-F03 |
| Ischemic heart disease (1) | I20-I25 |
| Heart failure (1) | I50 |
| Cerebrovascular disease (1) | I60-I69 |
| Vascular diseases (1) | I70-I79 |
| Chronic pulmonary diseases (1) | J43-J46 |
| Chronic liver disease (1) | K70-K77 |
| Tumor without metastasis (2) | C00-C76, C80, C97 |
| Lymphoma or leukemia (2) | C81-C96 |
| Kidney disease (1) | N17-N19 |
| moderate or severe (+1) | N18.2-N18.5 |
| Hemiplegia (2) | G81 |
| Peptic ulcer disease (2) | K25-K27 |
| Metastatic solid tumor (6) | C77-C79 |
| Osteoporosis diagnosis | M80-M81 |
| Conditions associated with osteoporosis | Hyperthyroidism (E05), Hypogonadism (E28-E29), Malnutrition (E40-E46), Osteogenesis imperfecta (Q780), Chronic liver disease (K70-K77), Hyperparathyroidism (E21) |
| Alcohol related disease | Mental and behavioral disorders due to use of alcohol (F10), Degeneration of nervous system due to alcohol (G312), Alcoholic polyneuropathy (G621), Alcoholic myopathy (G721), Alcoholic cardiomyopathy (I426), Alcoholic gastritis (K292), Alcoholic liver disease (K70), Alcohol-induced acute pancreatitis (K852), Alcohol-induced chronic pancreatitis (K860), Toxic effect of alcohol (T51) |
| Rheumatoid arthritis | M05-M06 |
| Osteoporosis medication | Prescriptions: Bisphosphonates (M05BA and M05BB), Denosumab (M05BX04), Teriparatide (H05AA02), Strontium (M05BX03), Raloxifene (G03XC01), Testosterone (G03BA03), Systemic estrogens (G03CA) Tibolone (G03CX01). |
|  | Non-prescribed parenteral treatment offered to patients at outpatient clinics or while admitted: The combination of an osteoporosis diagnosis (M80, M81 or M859) and a code for intravenous (DT016) or subcutaneous (DT021) administration was used. |
| Calcium + Vitamin D | ATC-code A12AX, repeated (≥2 prescriptions last year) and recent (last prescription within 120 days) |
| Oral prednisolone | Any previous three-month period in which more than 450 mg prednisolone (ATC-code H02AB06) were collected, i.e. more than 5 mg/day within a three-month period |
| Prevalent fracture | Any previous fracture (see outcomes for definitions) |
| Prevalent fall injury | Any previous non-skeletal fall injury resulting in a hospital visit or admission.  W00-W19 code and a S00-T14 diagnosis, but not a simultaneous fracture code. |
|  |  |

| **Variable (continued)** | **Definitions using ICD-10 Codes and/or ATC-Codes** |
| --- | --- |
| Cardiovascular medications | Repeated (≥2 collected prescriptions last year) and Recent (last prescription within 120 days) |
| Nitrates | ATC-code C01DA |
| Diuretics | ATC-code C03B, C03C, C03D, C03E, C03X |
| Thiazides | ATC-code C03A |
| Beta blockers | ATC-code C07 |
| Calcium channel blockers, | ATC-code C08 |
| RAS inhibitors | ATC-code C09 |
| Statins | ATC-code C10AA |
|  |  |
| T2DM medications | Any collected prescriptions last year |
| Any | ATC-code A10 |
| Insulin | ATC-code A10A |
| Metformin | ATC-code A10BA |
| Sulfonylureas | ATC-code A10BB |
| DPP-4 inhibitors | ATC-code A10BH, A10BD07, A10BD08, A10BD10 or A10BD11 |
| GLP-1 analogues | ATC-code A10BJ |
| SGLT2 inhibitors, | ATC-code A10BK, A10BD15, A10BD16 or A10BD20 |
| Glitazones | ATC-code A10BG or A10BD05 |
|  |  |
| Information from Diabetes Register |  |
| Body mass index, kg/m^2^ | Without outdoor clothes and shoes |
| Systolic blood pressure, mmHg | Sitting after 5 minutes of rest |
| Diastolic blood pressure, mmHg | Sitting after 5 minutes of rest |
| Glycated hemoglobin, mmole/mole |  |
| Glycated hemoglobin, percent | Based on values from the National Glucohemoglobin Standardization Program |
| Cholesterol, total, mmole/liter |  |
| Age at diagnosis of diabetes | Gestational diabetes not included |
| Duration of diabetes at baseline | Gestational diabetes not included |
| Current smoker | 1 cigarette per day or more or pipe smoker. Includes those who stopped smoking less than three months earlier |
| Physical activity | 30 minutes’ walk or equivalent. Individual assessment of all forms of activities. |
| Chronic Kidney Disease (Renal failure) | Groups based on estimated GFR (ml/min/1.73 m^2^) calculated using the Modification of Diet in Renal Disease (MDRD) equation. |
|  |  |
|  |  |
|  |  |
