# Supplementary material for: Risk of fracture in adults with type 2 diabetes in Sweden: A national cohort study
Source: PLoS Med. 2023 Jan 26;20(1):e1004172. doi: 10.1371/journal.pmed.1004172 (PMC9910793; doi:10.1371/journal.pmed.1004172)
Supplement: S3 Table — Subgroup analyses per sex. Outcomes for patients with type 2 diabetes and population controls without diabetes, matched according to birth year, sex, and county. Event rates were calculated as the number of persons with respective outcome per 1,000 person-years and are presented with exact Poisson 95% CIs. The adjusted Cox model is adjusted for age, sex, sickness benefits, marital status, urban residency, non-Nordic citizenship at birth, Charlson comorbidity index, osteoporosis diagnosis, conditions associated with osteoporosis, alcohol-related disease, rheumatoid arthritis, osteoporosis medication, calcium + vitamin D, oral prednisolone, prevalent fracture, prevalent fall injury, nitrates, diuretics, thiazides, beta blockers, calcium channel blockers, RAS inhibitors, and statins. (DOCX) [file pmed.1004172.s014.docx]

## S3 Table: Outcomes for T2DM Patients vs. Controls According to Sex

|  |  |  |  |  |
| --- | --- | --- | --- | --- |
|  | **Men** | | **Women** | |
|  | **Controls** | **T2DM** | **Controls** | **T2DM** |
| **N=** | **327,101** | **327,101** | **253,026** | **253,026** |
|  |  |  |  |  |
| Time at risk, years median (IQR) | 6.3 (2.9-9.7) | 6.5 (3.1-9.7) | 6.8 (3.2-10.0) | 6.7 (3.2-9.8) |
|  |  |  |  |  |
| **Any fracture** |  |  |  |  |
| n (%) | 28,091 (4.8%) | 31,225 (5.4%) | 43,455 (7.5%) | 44,277 (7.6%) |
| Rate, per 1000 person-years | 14.5 (14.4-14.7) | 16.1 (15.9-16.2) | 29.7 (29.4-30.0) | 30.3 (30.0-30.6) |
| Cox, unadjusted, HR (95%CI) | Ref. [1] | 1.11 (1.09-1.13) | Ref. [1] | 1.02 (1.01-1.04) |
| Cox, adjusted, HR (95%CI) | Ref. [1] | 1.10 (1.08-1.12) | Ref. [1] | 1.05 (1.03-1.06) |
|  |  |  |  |  |
| **Major osteoporotic fracture** |  |  |  |  |
| n (%) | 15,218 (2.6%) | 16,496 (2.8%) | 30,809 (5.3%) | 30,399 (5.2%) |
| Rate, per 1000 person-years | 7.67 (7.55-7.79) | 8.24 (8.11-8.36) | 20.3 (20.1-20.6) | 20.0 (19.8-20.3) |
| Cox, unadjusted, HR (95%CI) | Ref. [1] | 1.08 (1.05-1.10) | Ref. [1] | 0.99 (0.97-1.00) |
| Cox, adjusted, HR (95%CI) | Ref. [1] | 1.09 (1.07-1.12) | Ref. [1] | 1.02 (1.01-1.04) |
|  |  |  |  |  |
| **Hip fracture** |  |  |  |  |
| n (%) | 7,278 (1.3%) | 7,720 (1.3%) | 12,219 (2.1%) | 12,985 (2.2%) |
| Rate, per 1000 person-years | 3.62 (3.53-3.70) | 3.80 (3.71-3.88) | 7.68 (7.55-7.82) | 8.20 (8.06-8.34) |
| Cox, unadjusted, HR (95%CI) | Ref. [1] | 1.05 (1.02-1.09) | Ref. [1] | 1.07 (1.04-1.10) |
| Cox, adjusted, HR (95%CI) | Ref. [1] | 1.09 (1.05-1.13) | Ref. [1] | 1.12 (1.09-1.15) |
|  |  |  |  |  |
| **Death** |  |  |  |  |
| n (%) | 56,127 (9.7%) | 79,728 (13.7%) | 48,018 (8.3%) | 65,500 (11.3%) |
| Rate, per 1000 person-years | 27.7 (27.4-27.9) | 38.9 (38.6-39.2) | 29.6 (29.3-29.8) | 40.5 (40.2-40.8) |
| Cox, unadjusted, HR (95%CI) | Ref. [1] | 1.41 (1.39-1.42) | Ref. [1] | 1.37 (1.36-1.39) |
| Cox, adjusted, HR (95%CI) | Ref. [1] | 1.31 (1.30-1.33) | Ref. [1] | 1.31 (1.29-1.33) |
|  |  |  |  |  |

Subgroup analyses per sex. Outcomes for patients with type 2 diabetes and population controls without diabetes, matched according to birth year, sex and county. Event rates were calculated as the number of persons with respective outcome per 1000 person-years and are presented with exact Poisson 95% confidence intervals. The adjusted Cox model is adjusted for age, gender, sickness benefits, marital status, urban residency, non-Nordic citizenship at birth, Charlson comorbidity index, osteoporosis diagnosis, conditions associated with osteoporosis, alcohol related disease, rheumatoid arthritis, osteoporosis medication, calcium + vitamin D, oral prednisolone, prevalent fracture, prevalent fall injury, nitrates, diuretics, thiazides, beta blockers, calcium channel blockers, RAS inhibitors and statins.
