# Supplementary material for: Risk of fracture in adults with type 2 diabetes in Sweden: A national cohort study
Source: PLoS Med. 2023 Jan 26;20(1):e1004172. doi: 10.1371/journal.pmed.1004172 (PMC9910793; doi:10.1371/journal.pmed.1004172)
Supplement: S4 Table — (DOCX) [file pmed.1004172.s015.docx]

## S4 Table: Baseline Characteristics of Patients with Type 2 Diabetes and Controls According to Sex

|  |  |  |  |  |  |  |  |
| --- | --- | --- | --- | --- | --- | --- | --- |
|  | **Male Controls** | **Male T2DM** |  |  | **Female Controls** | **Female T2DM** |  |
| **A. Information from National Registers** | **N=327,101** | **N=327,101** | **SMD*** |  | **N=253,026** | **N=253,026** | **SMD** |
| Age, years, mean (SD) | 65.46 (11.46) | 65.46 (11.46) | <0.001 |  | 68.30 (12.31) | 68.30 (12.31) | <0.001 |
| Sickeness benefits, n (%) | 16,049 4.9) | 26,757 8.2) | 0,133 |  | 14,344 5.7) | 19,816 7.8) | 0,086 |
| Marital status |  |  | 0,077 |  |  |  | 0,058 |
| Married, n (%) | 198,303 60.6) | 186,062 56.9) |  |  | 121,537 48.0) | 114,975 45.4) |  |
| Unmarried, n (%) | 57,338 17.5) | 63,458 19.4) |  |  | 29,520 11.7) | 29,189 11.5) |  |
| Divorced, n (%) | 50,898 15.6) | 55,682 17.0) |  |  | 44,036 17.4) | 46,141 18.2) |  |
| Widow(er), n (%) | 20,562 6.3) | 21,899 6.7) |  |  | 57,933 22.9) | 62,721 24.8) |  |
| Urban recidency, (>200 per km^2^), n (%) | 83,272 25.5) | 81,312 24.9) | 0,014 |  | 65,215 25.8) | 62,247 24.6) | 0,027 |
| Non-nordic citizenship at birth, n (%) | 24,101 7.4) | 38,634 11.8) | 0,151 |  | 17,490 6.9) | 30,762 12.2) | 0,179 |
| Charlson comorbidity index |  |  | 0,254 |  |  |  | 0.230 |
| 0, n (%) | 247,737 (75.7) | 212,860 (65.1) |  |  | 197,678 (78.1) | 173,748 (68.7) |  |
| 1-2, n (%) | 63,179 (19.3) | 88,762 (27.1) |  |  | 45,559 (18) | 63,158 (25) |  |
| ≥3, n (%) | 16,185 (4.9) | 25,479 (7.8) |  |  | 9,789 (3.9) | 16,120 (6.4) |  |
| Osteoporosis diagnosis, n (%) | 1,032 0.3) | 910 0.3) | 0,007 |  | 6,470 2.6) | 4,537 1.8) | 0,052 |
| Conditions associated with osteoporosis, n (%)^†^ | 1,422 0.4) | 2,178 0.7) | 0,031 |  | 3,346 1.3) | 4,776 1.9) | 0,045 |
| Alcohol related disease, n (%) | 6,470 2.0) | 9,186 2.8) | 0,054 |  | 1,796 0.7) | 2,627 1.0) | 0,035 |
| Rheumatoid arthritis, n (%) | 2,454 0.8) | 2,805 0.9) | 0,012 |  | 4,538 1.8) | 4,679 1.8) | 0,004 |
| Osteoporosis medication, n (%) | 2,647 0.8) | 3,130 1.0) | 0,016 |  | 16,944 6.7) | 13,160 5.2) | 0,063 |
| Calcium + Vitamin D, n (%) | 4,035 1.2) | 4,721 1.4) | 0,018 |  | 18,512 7.3) | 16,622 6.6) | 0,029 |
| Oral prednisolone, n (%) | 14,152 4.3) | 17,430 5.3) | 0,047 |  | 14,674 5.8) | 19,365 7.7) | 0,074 |
| Prevalent fracture, n (%) | 35,704 10.9) | 36,299 11.1) | 0,006 |  | 43,608 17.2) | 40,874 16.2) | 0,029 |
| Prevalent fall injury, n (%) | 28,408 8.7) | 30,901 9.4) | 0,027 |  | 21,982 8.7) | 24,381 9.6) | 0,033 |
| Nitrates, n (%) | 9,861 3.0) | 20,834 6.4) | 0,159 |  | 7,856 3.1) | 16,615 6.6) | 0,162 |
| Diuretics, n (%) | 32,109 9.8) | 66,988 20.5) | 0,301 |  | 41,455 16.4) | 76,946 30.4) | 0,336 |
| Thiazides, n (%) | 10,136 3.1) | 17,674 5.4) | 0,114 |  | 12,538 5.0) | 19,240 7.6) | 0,109 |
| Beta blockers, n (%) | 60,117 18.4) | 115,211 35.2) | 0,387 |  | 51,001 20.2) | 93,656 37.0) | 0.380 |
| Calcium channel blockers, , n (%) | 34,607 10.6) | 71,947 22.0) | 0,313 |  | 26,540 10.5) | 53,233 21.0) | 0,293 |
| RAS inhibitors, n (%) | 64,167 19.6) | 151,627 46.4) | 0,593 |  | 43,238 17.1) | 107,781 42.6) | 0,58 |
| Statins, n (%) | 48,555 14.8) | 121,698 37.2) | 0,527 |  | 30,280 12.0) | 86,575 34.2) | 0,547 |

|  | **Male Controls** | **Male T2DM** |  |  | **Female Controls** | **Female T2DM** |  |
| --- | --- | --- | --- | --- | --- | --- | --- |
| **A. Information from National Registers (cont.)** | **N=327,101** | **N=327,101** | **SMD*** |  | **N=253,026** | **N=253,026** | **SMD** |
| T2DM medications any, n (%) | 0 0.0) | 209,229 64.0) | 1,884 |  | 0 0.0) | 150,889 59.6) | 1,719 |
| Insulin, n (%) | 0 0.0) | 62,974 19.3) | 0,691 |  | 0 0.0) | 45,509 18.0) | 0,662 |
| Metformin, n (%) | 0 0.0) | 157,722 48.2) | 1,365 |  | 0 0.0) | 112,917 44.6) | 1.270 |
| Sulfonylureas, n (%) | 0 0.0) | 44,469 13.6) | 0,561 |  | 0 0.0) | 33,142 13.1) | 0,549 |
| DPP-4 inhibitors, n (%) | 0 0.0) | 4,496 1.4) | 0,167 |  | 0 0.0) | 2,726 1.1) | 0,148 |
| GLP-1 analoges, n (%) | 0 0.0) | 884 0.3) | 0,074 |  | 0 0.0) | 556 0.2) | 0,066 |
| SGLT2 inhibitors, , n (%) | 0 0.0) | 400 0.1) | 0,049 |  | 0 0.0) | 190 0.1) | 0,039 |
| Glitazones, n (%) | 0 0.0) | 5,723 1.7) | 0,189 |  | 0 0.0) | 3,924 1.6) | 0,177 |
|  |  |  |  |  |  |  |  |
| **B. Information from Diabetes Register** | **Controls** | **T2DM** | **N (%)** |  | **Controls** | **T2DM** | **N (%)** |
| Body mass index (BMI), kg/m^2^ | – | 29.9±6.8 | 238,442 (73%) |  | – | 30.6±7.5 | 177,845 (70%) |
| Normal/underweight (<25), n (%) |  | 35,632 (15.0) |  |  |  | 30,793 (17.3) |  |
| Overweight (25-29.9), n (%) |  | 101,082 (42.4) |  |  |  | 60,246 (33.9) |  |
| Obesity class I (30-34.9), n (%) |  | 68,274 (28.7) |  |  |  | 50,120 (28.2) |  |
| Obesity class II (≥35), n (%) |  | 33,252 (14.0) |  |  |  | 36,584 (20.6) |  |
| Systolic blood pressure, mmHg | – | 137.3±16.7 | 258,435 (79%) |  | – | 138.5±17.6 | 196,375 (78%) |
| Diastolic blood pressure, mmHg | – | 79.2±10.2 | 258,141 (79%) |  | – | 77.5±9.9 | 196,111 (78%) |
| Glycated hemoglobin (HbA1c) |  |  | 269,277 (82%) |  |  |  | 205,060 (81%) |
| mmole/mole | – | 54.9±16.6 |  |  | – | 53.4±15.1 |  |
| % | – | 7.2±1.5 |  |  | – | 7.0±1.4 |  |
| Cholesterol, total, mmole/liter | – | 4.9±1.1 | 210,507 (64%) |  | – | 5.2±1.1 | 154,058 (61%) |
| Age at diagnosis of diabetes, years | – | 60.4±11.5 | 305,196 (93%) |  | – | 62.8±12.3 | 232,543 (92%) |
| Median duration of diabetes at baseline (IQR), years | – | 2 (0-8.1) | 305,196 (93%) |  | – | 2 (0-9) | 232,543 (92%) |
| Current smoking – no (%) | – | 36,095 (15.8) | 229,172 (70%) |  | – | 25,940 (14.9%) | 173,916 (69%) |
| Physical activity – no (%)**^§^** |  |  | 196,992 (60%) |  |  |  | 148,226 (59%) |
| Never | – | 28,540 (14.5) |  |  | – | 25,242 (17.0) |  |
| <1 per week | – | 25,870 (13.1) |  |  | – | 20,390 (13.8) |  |
| 1-2 per week | – | 39,399 (20.0) |  |  | – | 29,989 (20.2) |  |
| 3-5 per week | – | 43,474 (22.1) |  |  | – | 31,134 (21.0) |  |
| Daily | – | 59,709 (30.3) |  |  | – | 41,471 (28.0) |  |
| Chronic Kidney Disease (Renal failure) - no (%) |  |  | 240,445 (74%) |  |  |  | 183,166 (72%) |
| No (GFR ≥60) | – | 208,987 (86.9) |  |  | – | 142,521 (77.8) |  |
| Moderate (GFR 30-59.9) | – | 29,442 (12.2) |  |  | – | 38,268 (20.9) |  |
| Severe (GFR 15-29.9) | – | 1,682 (0.7) |  |  | – | 2,129 (1.2) |  |
| Terminal (GFR <15) | – | 334 (0.1) |  |  | – | 248 (0.1) |  |
