# Supplementary material for: Risk of fracture in adults with type 2 diabetes in Sweden: A national cohort study
Source: PLoS Med. 2023 Jan 26;20(1):e1004172. doi: 10.1371/journal.pmed.1004172 (PMC9910793; doi:10.1371/journal.pmed.1004172)
Supplement: S5 Table — Outcomes for patients with type 2 diabetes and population controls without diabetes, matched according to birth year, sex, and county. Event rates were calculated as the number of persons with respective outcome per 1,000 person-years and are presented with exact Poisson 95% CIs. The adjusted Cox model is adjusted for age, sex, sickness benefits, marital status, urban residency, non-Nordic citizenship at birth, Charlson comorbidity index, osteoporosis diagnosis, conditions associated with osteoporosis, alcohol-related disease, rheumatoid arthritis, osteoporosis medication, calcium + vitamin D use, oral prednisolone medication use, prevalent fracture, prevalent fall injury, nitrates, diuretics, thiazides, beta blockers, calcium channel blockers, renin-angiotensin system inhibitors, and statins. (DOCX) [file pmed.1004172.s016.docx]

## S5 Table: Other Outcomes for Patients with Type 2 Diabetes and Controls

|  |  |  |
| --- | --- | --- |
|  | **Controls** | **T2DM** |
| N= | 580,127 | 580,127 |
|  |  |  |
| Time at risk, years median (IQR) | 6.6 (3.0-9.8) | 6.6 (3.1-9.7) |
|  |  |  |
| **Lower Leg Fracture** |  |  |
| n (%) | 10645 (1.8%) | 12916 (2.2%) |
| Rate, per 1000 person-years | 3.0 (2.9-3.0) | 3.6 (3.5-3.6) |
| Cox, unadjusted, HR (95%CI) | Ref. [1] | 1.21 (1.18-1.24) |
| Cox, adjusted, HR (95%CI) | Ref. [1] | 1.18 (1.14-1.21) |
|  |  |  |
| **Wrist Fracture** |  |  |
| n (%) | 14505 (2.5%) | 11153 (1.9%) |
| Rate, per 1000 person-years | 4.0 (4.0-4.1) | 3.1 (3.0-3.1) |
| Cox, unadjusted, HR (95%CI) | Ref. [1] | 0.76 (0.74-0.78) |
| Cox, adjusted, HR (95%CI) | Ref. [1] | 0.80 (0.78-0.82) |
|  |  |  |
| **Proximal Humerus Fracture** |  |  |
| n (%) | 8827 (1.5%) | 10629 (1.8%) |
| Rate, per 1000 person-years | 2.4 (2.4-2.5) | 2.9 (2.9-3.0) |
| Cox, unadjusted, HR (95%CI) | Ref. [1] | 1.20 (1.17-1.24) |
| Cox, adjusted, HR (95%CI) | Ref. [1] | 1.21 (1.18-1.25) |
|  |  |  |
| **Injurious falls** |  |  |
| n (%) | 57,952 (10.0%) | 69,089 (11.9%) |
| Rate, per 1000 person-years | 16.8 (16.6-16.9) | 20.1 (20.0-20.3) |
| Cox, unadjusted, HR (95%CI) | Ref. [1] | 1.20 (1.19-1.22) |
| Cox, adjusted, HR (95%CI) | Ref. [1] | 1.18 (1.16-1.19) |
|  |  |  |

Outcomes for patients with type 2 diabetes and population controls without diabetes, matched according to birth year, sex and county. Event rates were calculated as the number of persons with respective outcome per 1000 person-years and are presented with exact Poisson 95% confidence intervals. The adjusted Cox model is adjusted for age, gender, sickness benefits, marital status, urban residency, non-Nordic citizenship at birth, Charlson comorbidity index, osteoporosis diagnosis, conditions associated with osteoporosis, alcohol related disease, rheumatoid arthritis, osteoporosis medication, calcium + vitamin D use, oral prednisolone medication use, prevalent fracture, prevalent fall injury, nitrates, diuretics, thiazides, beta blockers, calcium channel blockers, renin-angiotensin system (RAS) inhibitors and statins.
