# Supplementary material for: Risk of fracture in adults with type 2 diabetes in Sweden: A national cohort study
Source: PLoS Med. 2023 Jan 26;20(1):e1004172. doi: 10.1371/journal.pmed.1004172 (PMC9910793; doi:10.1371/journal.pmed.1004172)
Supplement: S6 Table — Subdistribution hazard ratios with 95% CI for T2DM vs. controls for fractures and injurious falls. Calculated in a subset of 50.000 randomly selected persons using a Fine and Grey model with death as the competing risk. (DOCX) [file pmed.1004172.s017.docx]

## S6 Table: Subdistribution hazard ratios for T2DM Patients vs. Population Controls with Consideration of Competing Risk of Death

| **Event** | **SHR (95%CI)** |  |
| --- | --- | --- |
| Any fracture | 0.98 (0.94-1.03) |  |
| Major osteoporotic fracture | 0.96 (0.90-1.02) |  |
| Hip fracture | 0.99 (0.90-1.09) |  |

Subhazard ratios (SHR) with 95% CI for T2DM vs controls for fractures and injurious falls. Calculated in a subset of 50.000 randomly selected persons using a Fine & Grey model with death as the competing risk.

## Figure S5a: Identification of Diabetes Related Risk Factors using Gradient Boosting Machines – all cases including imputed values

**
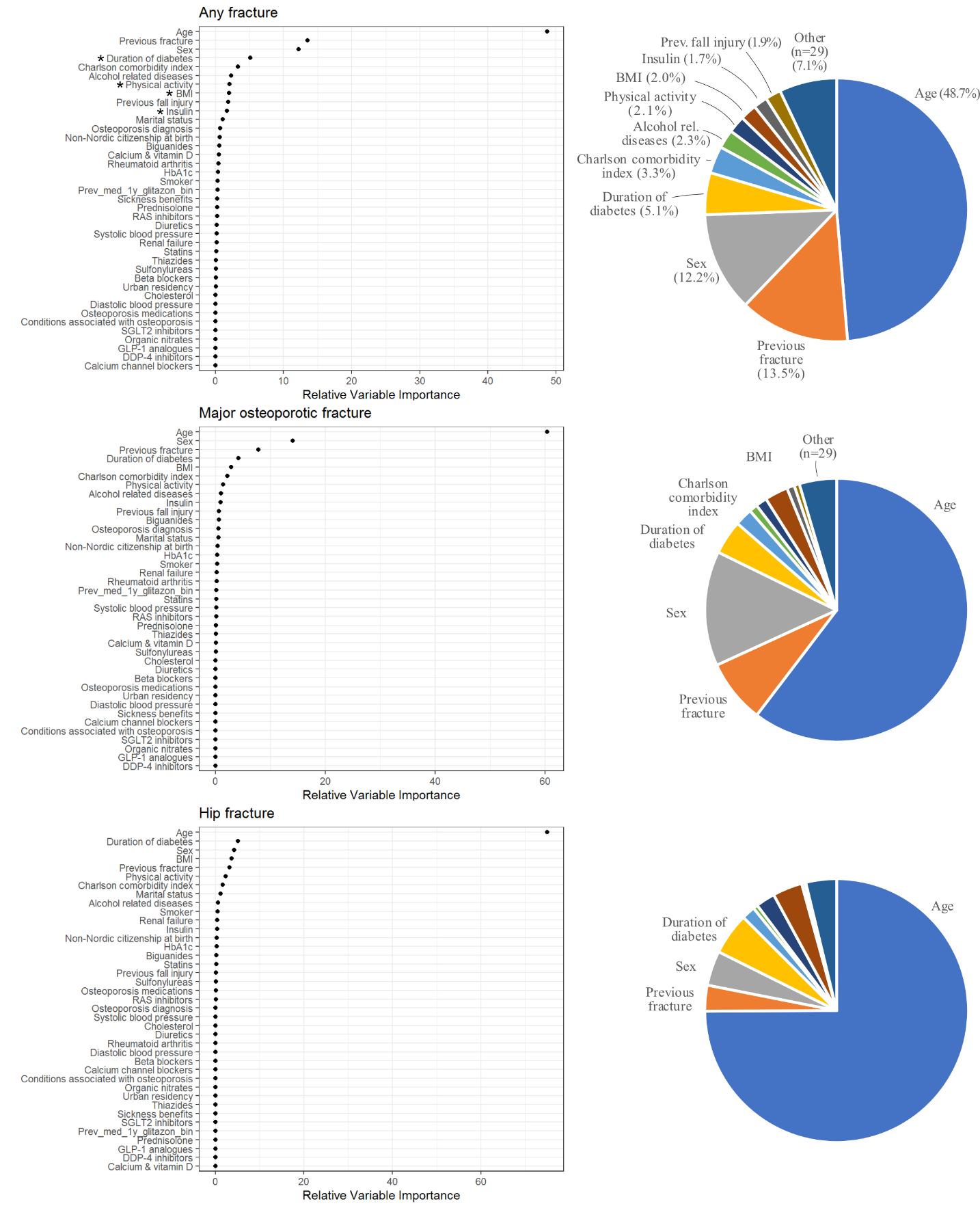
**

Machine learning using Gradient Boosting Machines was applied to all T2DM cases (N=580,127, no controls). The settings allowed interaction depth two, i.e. all pairwise interactions were enabled. All variables in Table 1, both general comorbidity and fracture risk factors as well as specific diabetes related variables (imputed) were included. For any fracture, the top four diabetes related variables are marked with an asterix and percentages for relative importance included in the pie chart. The color labels are the same for all three outcomes.

## Figure S5b: Identification of Diabetes Related Risk Factors Using Gradient Boosting Machines – only complete cases, no imputed values


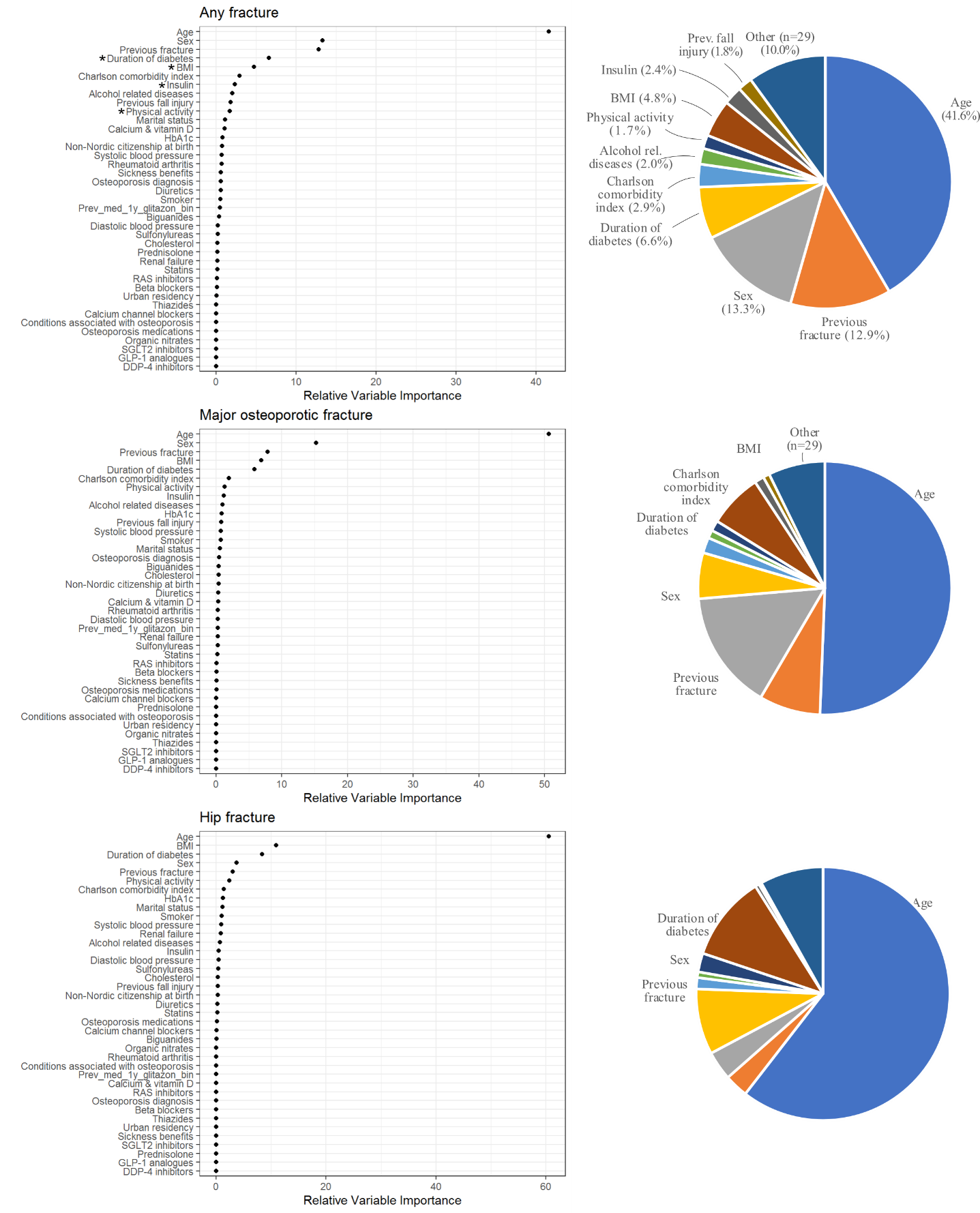


Machine learning using Gradient Boosting Machines was applied to all T2DM cases with complete values (N=209,802, no controls). The settings allowed interaction depth two, i.e. all pairwise interactions were enabled. All variables in Table 1, both general comorbidity and fracture risk factors as well as specific diabetes related variables were included. For any fracture, the top four diabetes related variables are marked with an asterix and percentages for relative importance included in the pie chart. The color labels are the same for all three outcomes.

## Figure S6 a-d: Analysis of the Top Four Variables Independent Association to Any Fracture


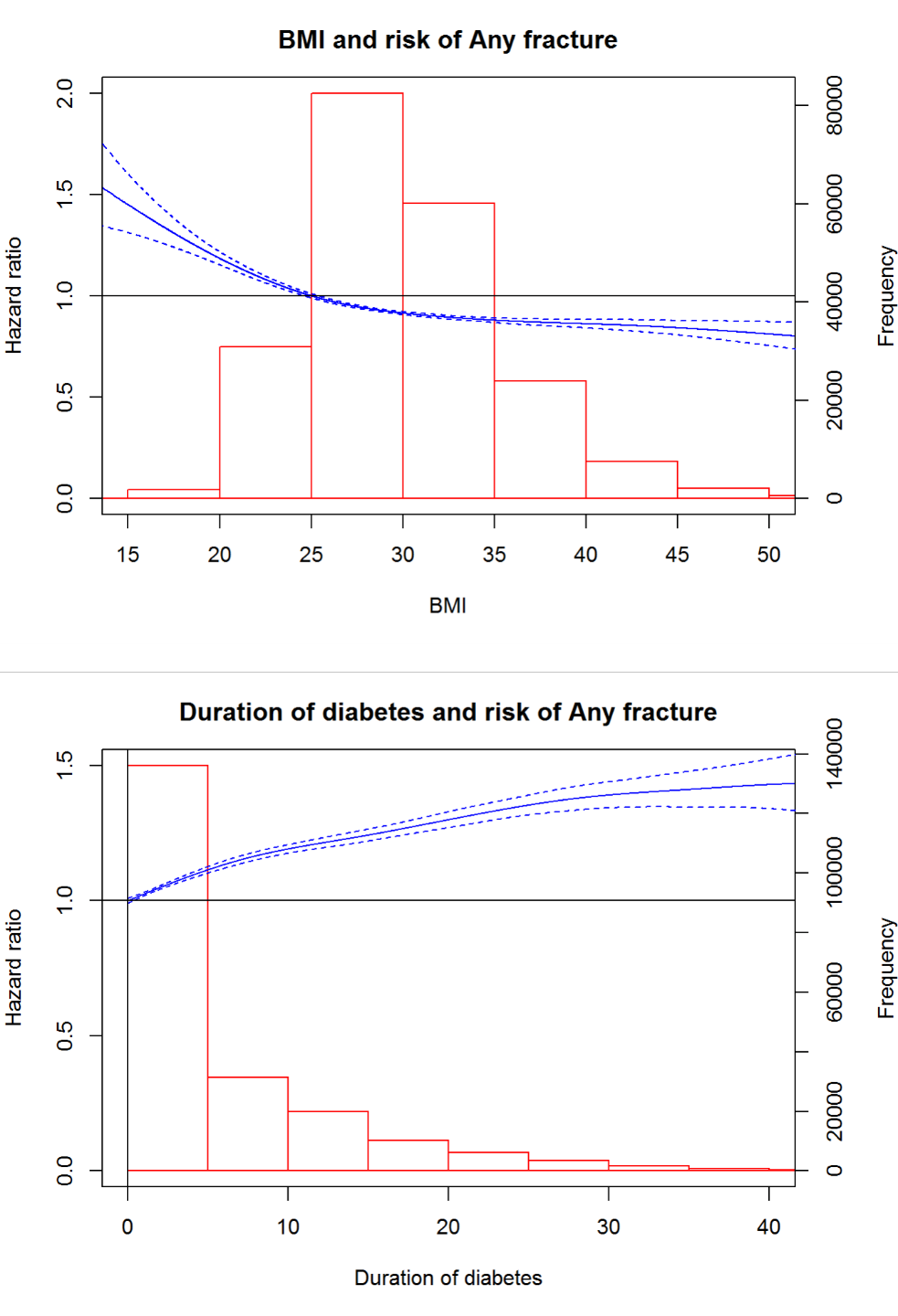

Number of T2DM patients are indicated on the left y-axis and the Hazard Ratios on the right y-axis.

All the T2DM cases (without controls) were included in a Cox regression model, fully adjusted and also including the variables from the Diabetes Register, to investigate the risk of any fracture. Imputed values from the Diabetes Register were included, the continuous variables were splined with five degrees of freedom. The figures illustrate the association between the top four covariates and any fracture with their respective histograms included. All values are from the same regression model. Number of T2DM patients are indicated on the left y-axis and the Hazard Ratios on the right y-axis.

## Figure S7 a-d: Comparison of T2DM Patients to Population Controls – Per Risk Factor

a. Risk of any fracture among T2DM patients compared to matched population controls. The analysis was stratified for BMI. Values of BMI were rounded off to integers. The risk of any fracture was analyzed using multivariable adjusted Cox models. A 20% risk increase (HR 1.20) was used as a clinically relevant threshold. Number of cases/controls in each group is indicated on the left y-axis and the Hazard Ratios on the right y-axis.

b. Risk of any fracture among T2DM patients compared to matched population controls. The analysis was stratified for duration of diabetes. Values of duration were rounded off to integers. The risk of any fracture was analyzed using multivariable adjusted Cox models. A 20% risk increase (HR 1.20) was used as a clinically relevant threshold. Number of cases/controls in each group is indicated on the left y-axis and the Hazard Ratios on the right y-axis.

c. Risk of any fracture among T2DM patients compared to matched population controls. Analysis stratified per level of physical activity. The risk of any fracture was analyzed using multivariable adjusted Cox models. A 20% risk increase (HR 1.20) was used as threshold for risk increase. Number of cases/controls in each group is indicated on the left y-axis and the Hazard Ratios on the right y-axis.

d. Risk of any fracture among T2DM patients with or without insulin treatment last year compared to matched population controls. The risk of any fracture was analyzed using multivariable adjusted Cox models. A 20% risk increase (HR 1.20) was used as threshold for risk increase. Number of cases/controls in each group is indicated on the left y-axis and the Hazard Ratios on the right y-axis.

## Figure S8: Risk of Fracture in T2DM Patients Compared to Matched Controls - per Age and Number of Risk Factors
